# Supplementary material for: Structural Basis for Phosphorylation-Dependent Recruitment of Tel2 to Hsp90 by Pih1
Source: Structure. 2014 Jun 10;22(6):805–18. doi: 10.1016/j.str.2014.04.001 (PMC4058522; doi:10.1016/j.str.2014.04.001)
Supplement: Document S1. Figures S1–S6 [file mmc1.pdf]

Structure, Volume *22*

Supplemental Information

Structural Basis for Phosphorylation-Dependent Recruitment of Tel2 to Hsp90 by Pih1

Mohinder Pal, Marc Morgan, Sarah E.L. Phelps, S. Mark Roe, Sarah Parry-Morris, Jessica A. Downs, Sigrun Polier, Laurence H. Pearl, and Chrisostomos Prodromou

Figure S1 – related to Figure 2

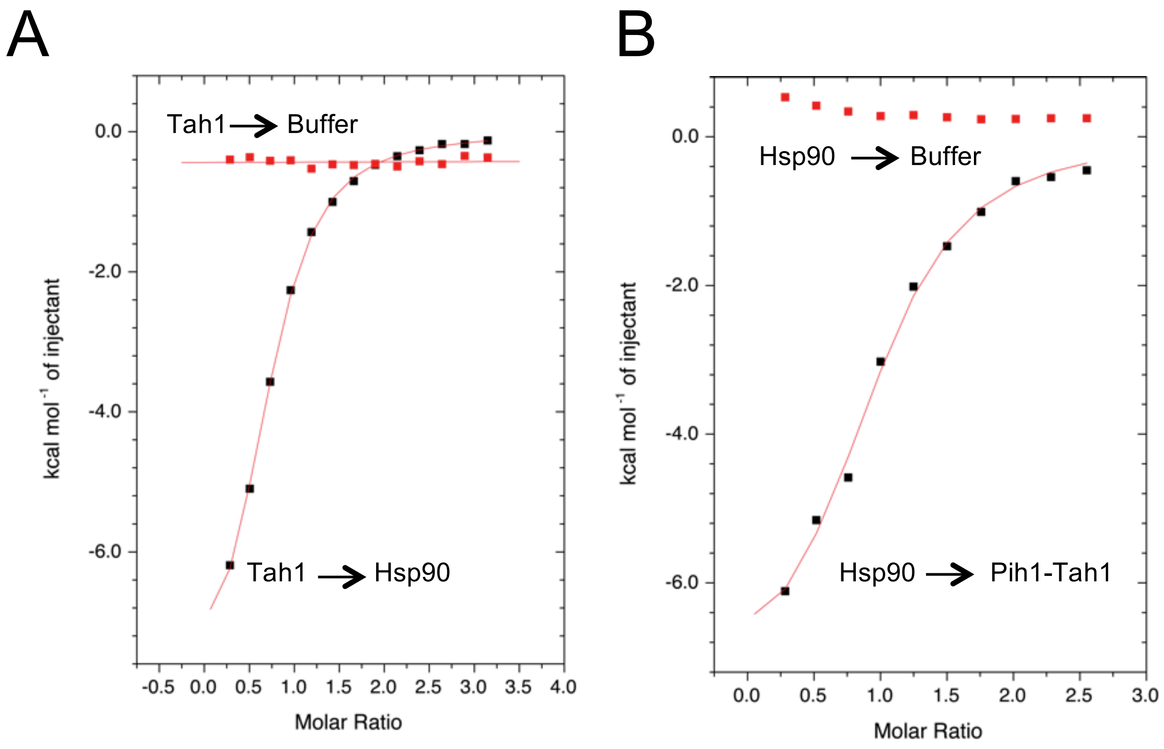

N = 0.68 ± 0.01 sites
K_D = 5.9 ± 0.30 μM
ΔH = -8445 ± 164.2 cal/mol
ΔS = -3.95 cal/mol/deg

N = 0.93 ± 0.04 sites
K_D = 4.0 ± 0.07 μM
ΔH = -7595 ± 404 cal/mol
ΔS = -0.367 cal/mol/deg

Pih1 interaction with Tah1 Dimer

A and B. Binding of Tah1 (**A**) or Tah1-Pih1 (**B**) to an Hsp90 dimer shows very similar affinity (K_D = 5.9 ± 0.3 μM, and K_D = 4 ± 0.07 μM, respectively) with a 1:1 stoichiometry (1 Hsp90 dimer: 2 Tah1) in both cases, confirming the possibility of each Tah1 domain bound to an Hsp90 dimer being able to recruit a Pih1 molecule.

Figure S2 – related to Figure 4

A

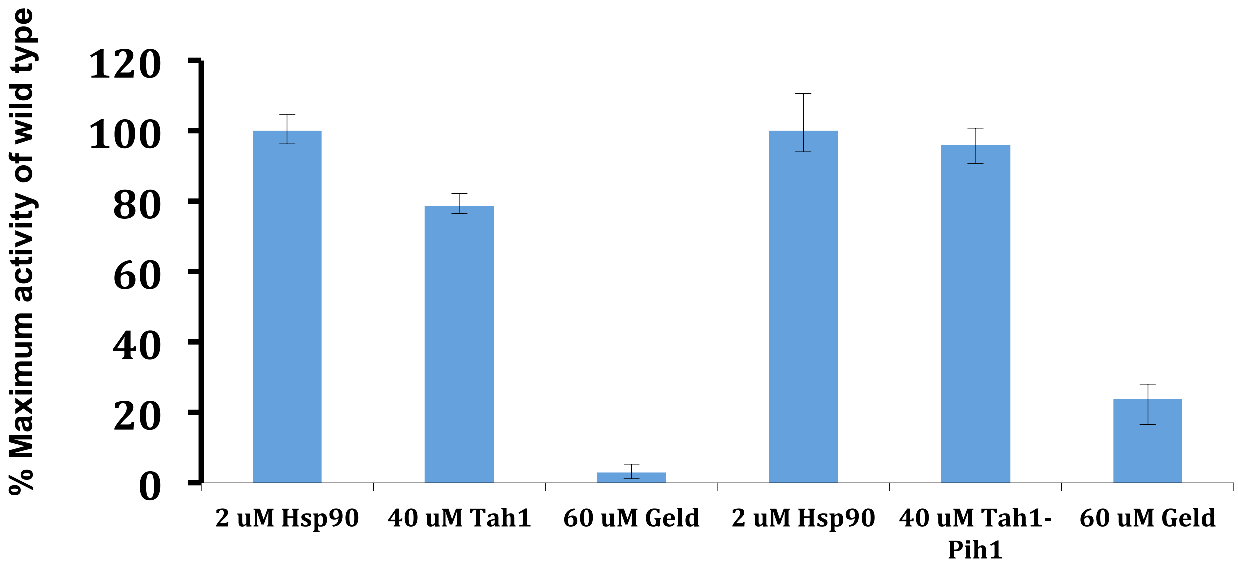

B

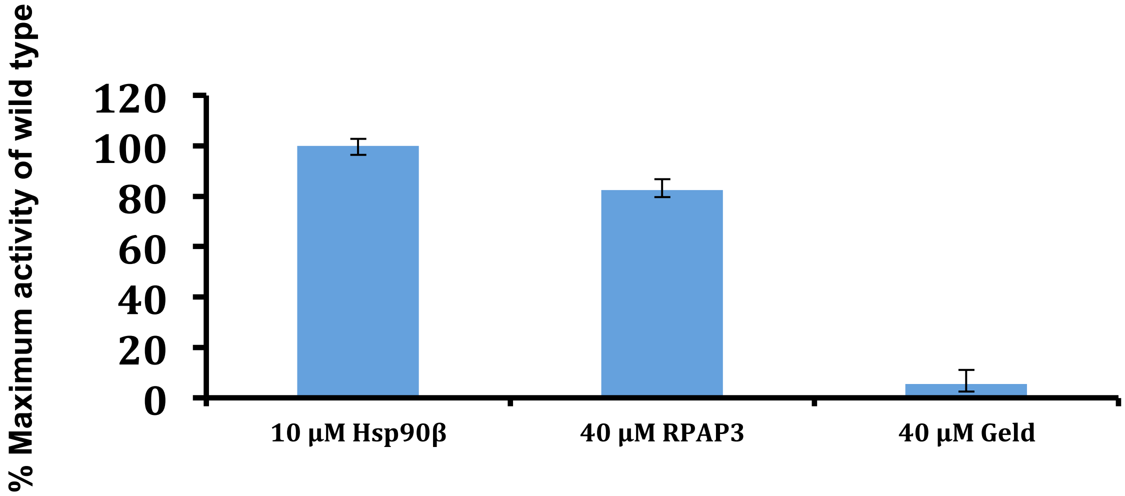

The effect of Tah1 and Spagh/RPAP3 binding on the ATPase activity of Hsp90

A. Binding of Tah1 and Tah1-Pih complex failed to show any strong regulatory effect on the ATPase activity of yeast Hsp90.

B. Binding of Spagh/RPAP3 failed to show any strong regulatory effect on the ATPase activity of human Hsp90β

Figure S3 – related to Figure 3

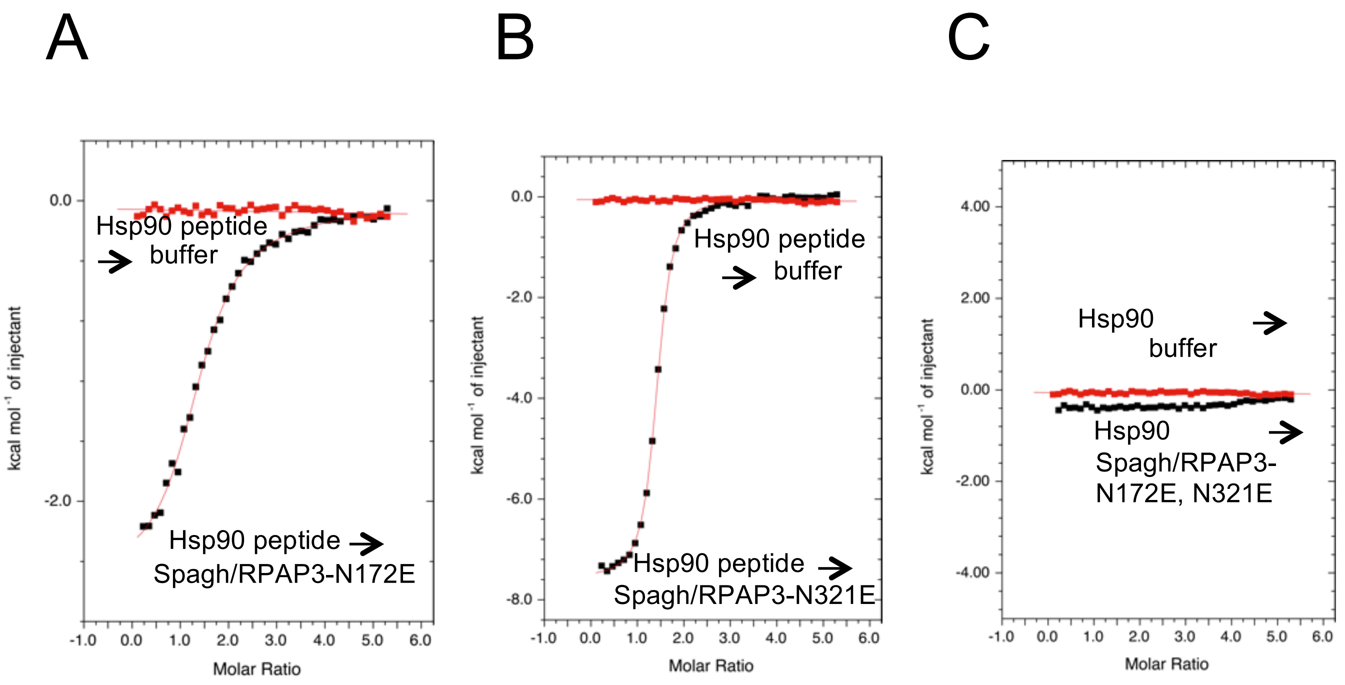

N1 = 1.36 ± 0.08 sites	N1 = 1.38 ± 0.02 sites
K_D1 = 9.7 ± 3.7 μM	K_D1 = 0.94 ± 0.94 μM
ΔH1 = -2500 ± 67.6 cal/mol	ΔH1 = -7584 ± 33.1 cal/mol
ΔS1 = 14.7 cal/mol/deg	ΔS1 = 2.55 cal/mol/deg
N2 = 1 site	N2 = 1 site
K_D2 = 231 ± 242 μM	K_D2 = 109.2 ± 270 μM
ΔH2 = -940.1 ± 172 cal/mol	ΔH2 = -100 ± 0.0 cal/mol
ΔS2 = 13.5 cal/mol/deg	ΔS2 = 17.8 cal/mol/deg

Binding of Hsp90 C-terminal peptide to Spagh/RPAP3 TPR domain mutants.

A. ITC of Hsp90 peptide binding to Spagh/RPAP3-N172E – TPR1 mutant. The single un-mutated site in TPR2 binds the Hsp90 peptide with K_D = 9.7 μM.

B. ITC of Hsp90 peptide binding to Spagh/RPAP3-N321E – TPR2 mutant. The single un-mutated site in TPR1 binds the Hsp90 peptide tightly with estimated K_D = 0.94 μM.

C. No Hsp90 peptide binding is observed with the Spagh/RPAP3-N172E, N321E double mutant.

Figure S4 – related to Figure 4

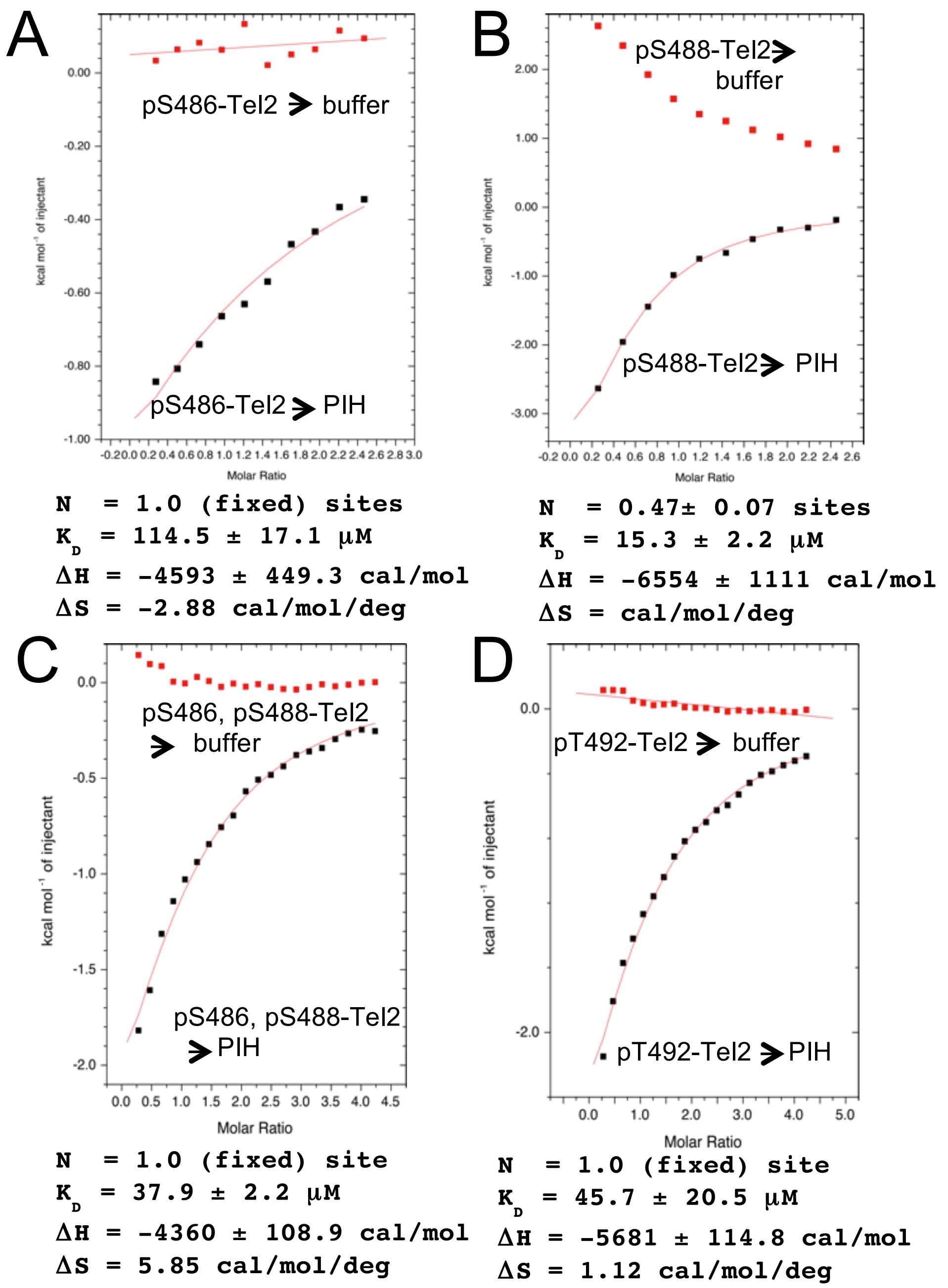

Binding of Tel2-peptides incorporating observed CK2 phosphorylation sites

A. ITC of binding of Tel2 peptide phosphorylated on Ser 486 to mouse Pih1-PIH domain. The peptide binds weakly with estimated K_D ~ 114.5 μM.

B. ITC of binding of Tel2 peptide phosphorylated on Ser 488 to mouse Pih1-PIH domain. The peptide binds with K_D = 15.3 μM.

C. ITC of binding of Tel2 peptide phosphorylated on Ser 486 and Ser 488 to mouse Pih1-PIH domain. The peptide binds with K_D = 37.9 μM.

D. ITC of binding of Tel2 peptide, where phosphorylated Ser 492 is substituted for phosphorylated Thr 492, to mouse Pih1-PIH domain. The peptide binds with K_D = 45.7 μM.

Figure S5 – related to Figure 5

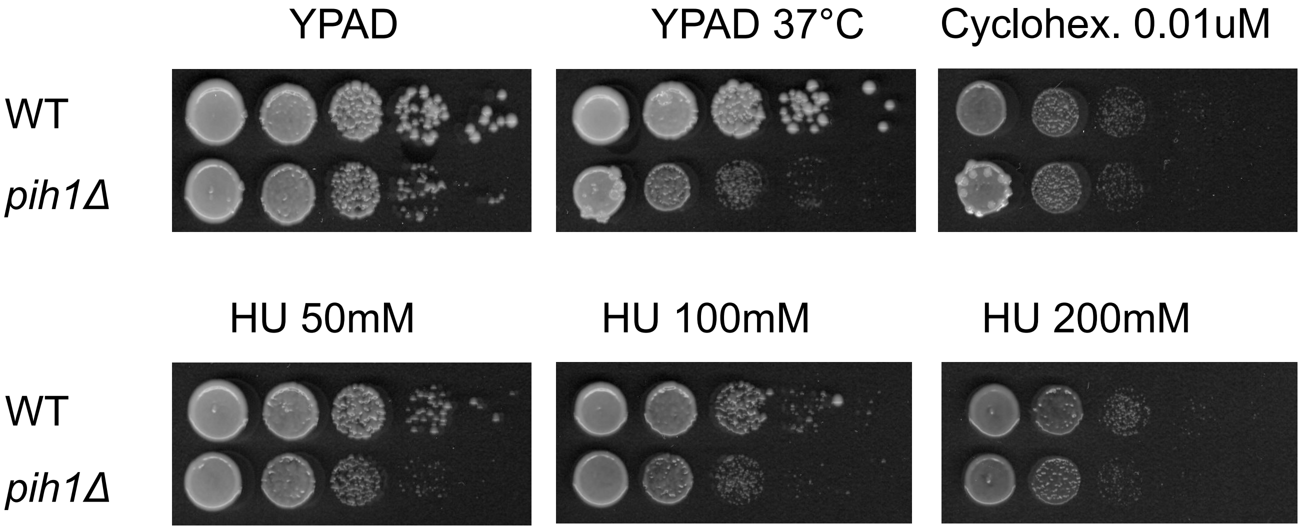

Pih1 and DNA damage sensitivity.

In contrast to the significant temperature sensitivity generated by the disruption of R2TP and TTT function, deletion of Pih1p does not confer any sensitivity to the DNA damaging agent hydroxyurea, compared to wild-type (WT).

Figure S6 – related to Figure 4

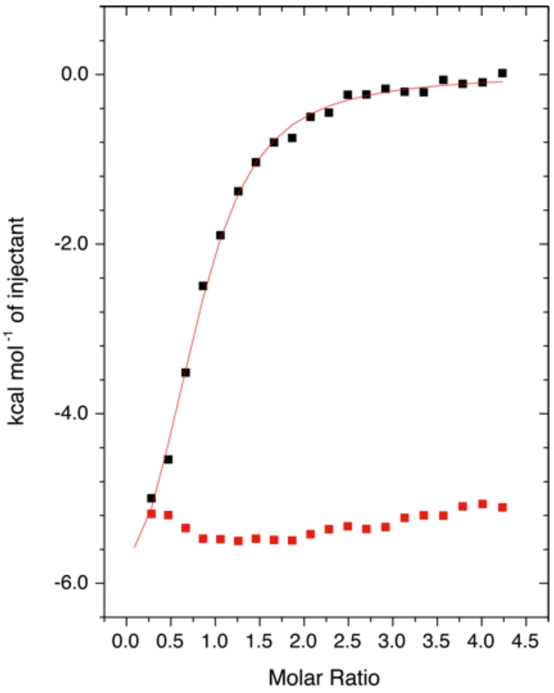

N = 0.76 ± 0.03 sites
K_D = 6.4 ± 0.7 μM
ΔH = -7313 ± 354.7 cal/mol
ΔS = -0.36 cal/mol/deg

The binding of the phosphoserine peptide of Mre11

A. Binding of the Mre11 phosphoserine peptide, MANDpSDDSI, to the PIH domain of Pih1. The peptide bound with a K_D = 6.4 μM.
