# Supplementary material for: Structural Basis for Phosphorylation-Dependent Recruitment of Tel2 to Hsp90 by Pih1
Source: Structure. 2014 Jun 10;22(6):805–18. doi: 10.1016/j.str.2014.04.001 (PMC4058522; doi:10.1016/j.str.2014.04.001)
Supplement: Document S2. Article plus Supplemental Information [file mmc3.pdf]

CellPress

Structural Basis for Phosphorylation-Dependent Recruitment of Tel2 to Hsp90 by Pih1

Mohinder Pal,[1,2] Marc Morgan,[1,2] Sarah E.L. Phelps,[1] S. Mark Roe,[1] Sarah Parry-Morris,[1] Jessica A. Downs,[1] Sigrun Polier,[1,4] Laurence H. Pearl,[1,3,*] and Chrisostomos Prodromou[1,3,*]
[1]MRC Genome Damage and Stability Centre, School of Life Sciences, University of Sussex, Falmer, Brighton BN1 9RQ, UK
[2]Co-first author
[3]Co-senior author
[4]Present address: Pharmaceutical Development, Sandoz GmbH, Biochemiestraße 10, 6336 Langkampfen, Austria
*Correspondence: laurence.pearl@sussex.ac.uk (L.H.P.), chris.prodromou@sussex.ac.uk (C.P.)
http://dx.doi.org/10.1016/j.str.2014.04.001
This is an open access article under the CC BY license (http://creativecommons.org/licenses/by/3.0/).

SUMMARY

Client protein recruitment to the Hsp90 system depends on cochaperones that bind the client and Hsp90 simultaneously and facilitate their interaction. Hsp90 involvement in the assembly of snoRNPs, RNA polymerases, PI3-kinase-like kinases, and chromatin remodeling complexes depends on the TTT (Tel2-Tti1-Tti2), and R2TP complexes—consisting of the AAA-ATPases Rvb1 and Rvb2, Tah1 (Spagh/RPAP3 in metazoa), and Pih1 (Pih1D1 in humans)—that together provide the connection to Hsp90. The biochemistry underlying R2TP function is still poorly understood. Pih1 in particular, at the heart of the complex, has not been described at a structural level, nor have the multiple protein-protein interactions it mediates been characterized. Here we present a structural and biochemical analysis of Hsp90-Tah1-Pih1, Hsp90-Spagh, and Pih1D1-Tel2 complexes that reveal a domain in Pih1D1 specific for binding CK2 phosphorylation sites, and together define the structural basis by which the R2TP complex connects the Hsp90 chaperone system to the TTT complex.

INTRODUCTION

The R2TP complex is implicated in the stabilization and assembly of an eclectic set of proteins and macromolecular complexes (Te et al., 2007; Zhao et al., 2005). These include RNA polymerase 2 (Boulon et al., 2010), small nucleolar ribonucleoproteins (snoRNPs; Kakihara and Houry, 2012), and phosphatidylinositol-3-kinase-like kinases (PIKKs) such as mTOR and SMG1 (Horejsí et al., 2010; Takai et al., 2010). The R2TP complex is found in organisms from yeast to humans, and consists of the AAA+ ATPases Rvb1 and Rvb2 (human: RUVBL1 and RUVBL2), a TPR-containing protein, Tah1 (human: Spagh or RPAP3), and Pih1 (also known as NOP17 and Pih1D1, but referred to as Pih1 henceforth; Kakihara and Houry, 2012; Te et al., 2007).

Pih1 is the multipoint scaffold of the R2TP complex, coupling the Rvb1-Rvb2 hetero-dodecamer, the Hsp90 chaperone machinery (via Tah1 in yeast or Spagh/RPAP3 in metazoa), and the TTT (Tel2-Tti1-Tti2) complex implicated in activation and stabilization of PIKKs (Hurov et al., 2010; Kakihara and Houry, 2012). Pih1 interaction with Rvb1-Rvb2 appears to be constitutive and direct and requires a central region of Pih1 (Paci et al., 2012), whereas the interaction of Pih1 with TTT is mediated by a casein kinase 2 (CK2) phosphorylated motif in Tel2 and an N-terminal region of Pih1 (Horejsí et al., 2010).

The biological roles ascribed to the R2TP complex are directly associated with its ability to interact with the Hsp90 chaperone system and function as an Hsp90 cochaperone (Boulon et al., 2010; Izumi et al., 2012; Takai et al., 2010). In yeast, recruitment of R2TP to Hsp90 is mediated by Tah1, a TPR-domain protein that simultaneously binds the conserved C-terminal MEEVD motif of Hsp90 and a C-terminal region of Pih1 (Eckert et al., 2010). In metazoa, the interaction of Pih1 and Hsp90 is mediated by Spagh/RPAP3, a much larger protein containing tandem TPR domains as well as additional domains of unknown function.

Whereas the R2TP complex is implicated in a growing number of biological processes, the mechanistic biochemistry underlying its function is poorly understood (Kakihara and Houry, 2012). Pih1, the core scaffold protein of the complex, has not been characterized structurally, nor have the protein-protein interactions it mediates been described at a structural level. Here we present crystal structures of Hsp90-Tah1-Pih1, Hsp90-Spagh/RPAP3, and Pih1-Tel2 complexes that together define key structural links within the R2TP core in yeast and in animals (Figure 1) and reveal how R2TP connects the Hsp90 chaperone system to the TTT complex involved in PIKK activation.

RESULTS

Structure of the Tah1-Pih1 Complex

We determined the crystal structure of full-length yeast Tah1, bound to both the C-terminal segment of yeast Pih1$^{264–344}$ and the C-terminal tail peptide (SRMEEVD) of Hsp90 (Figure 2A), by single-wavelength anomalous dispersion phasing of crystals grown with selenomethionine-labeled Tah1 (see Experimental Procedures) and refined to 2.2 Å (Table 1).

Consistent with earlier nuclear magnetic resonance studies (Back et al., 2013; Jiménez et al., 2012), the core of Tah1 is a TPR domain, which consists of α helices arranged in a repeating antiparallel right-handed helix topology. Unusually, Tah1-TPR contains five helices rather than the seven found in other

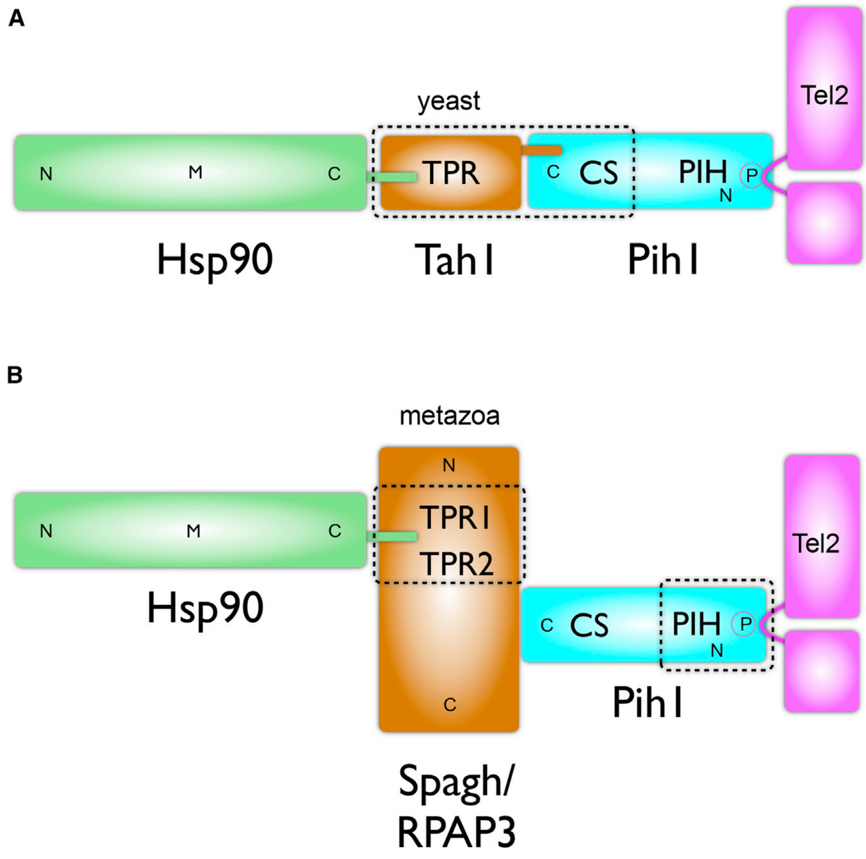

Figure 1. Hsp90-R2TP-Tel2 Connections

(A) Schematic of the chain of molecular interactions that connect the Hsp90 chaperone system to the Tel2 component of the TTT complex in yeast. Dotted lines represent interactions structurally defined in this study. N, M, and C represent N-, middle, and C-domains, respectively. TPR, tetratricopeptide repeat domain; CS, CHORD domain-containing protein and Sgt1 domain; and PIH, protein interacting with Hsp90 domain.

(B) As in (A), but for the metazoan R2TP system in which the role of Tah1 in yeast is fulfilled by Spagh/RPAP3. Dotted lines represent interactions structurally defined in this study.

structurally characterized Hsp90-binding TPR domains (Morgan et al., 2012; Scheufler et al., 2000; Zhang et al., 2005). The C-terminal 20 residues of Tah1 beyond the end of the fifth α helix of the TPR domain has no coherent structure in the isolated protein in solution (Back et al., 2013; Jiménez et al., 2012), but is fully ordered in the complex crystal structure, and forms the majority of the interaction with Pih1 (see below). The C-terminal domain of Pih1$^{264–344}$ consists of a seven-stranded β sandwich with the topology of a CS domain—a structural motif also found in Hsp90 cochaperones such as p23/Sba1 (Ali et al., 2006) and Sgt1 (Zhang et al., 2008).

Tah1-Pih1 Interaction

The Tah1-Pih1-CS interaction buries ~2,036 Å^2 of molecular surface, which is in the range typically associated with constitutive interactions (Krissinel and Henrick, 2007). Most (~1,720 Å^2) of this surface is buried by the C-terminal segment of Tah1 (Figure 2B). This segment snakes across one side of the Pih1-CS domain, extending the three-stranded β sheet via main-chain interactions between Ser 93 and Val 94 of Tah1 and Pro 299, Ser 300, Tyr 301, and Phe 303 of Pih1, before crossing over to extend the four-stranded β sheet via interactions between Val 99 and Val 101 of Tah1, and Ile 331, Phe 332, and His 333 of Pih1 (Figures 2B and 2C). These main-chain interactions are bolstered by packing of the side chains of Tah1 residues Val 94, Ile 96, Val 98, and Val 101 into a shallow hydrophobic channel formed by the side chains of Pih1 residues 297–303 and 326–334 on the edges of the stacked β sheets, with a single polar side chain interaction between Tah1-Glu 100 and Pih1-His 333 (Figure 2C).

Beyond the end of the β strand interaction at Val 101, Tah1 makes a right-angle bend at Asp 102 and Glu 103, packing the side chains of Leu 104 and Tyr 108 into a hydrophobic recess on the face of the four-stranded β sheet of Pih1, formed by the side chains of Ile 334, Phe 332, Tyr 341, and Tyr 343 (Figure 2D). A further bend in the trajectory of Tah1 forms a network of intramolecular polar and water-bridged interactions centered on Tah1-Arg 110 and delivers the carboxyl side chain of Asp 109 and the side chain and C-terminal α-carboxyl of Ser 111 into a polar/ion pair interaction with the basic side chains of Lys 272 and Arg 282 of Pih1 (Figure 2D).

The globular regions of the two proteins interact directly via a hydrophobic interface involving the packing of Pih1-Phe 303 into a pocket formed by Tah1 residues Thr 55, Ile 58, Val 91, Gly 92, and Val 94. This is reinforced by a bidentate hydrogen bonding interaction between the side chains of Tah1-Arg 66 and Pih1-Asp 328, and a well-ordered water network (Figure 2E). Whereas the extended C terminus of Tah1 dominates the interface with Pih1, these additional interactions fix the relative position of the two domains into a single globular entity.

Tah1 Interaction with the Conserved Hsp90-MEEVD Motif

The Tah1-Pih1-CS heterodimer was cocrystallized with a peptide incorporating the conserved C-terminal MEEVD motif found at the C terminus of all eukaryotic cytoplasmic Hsp90s and is essential for Hsp90 interaction with TPR-domain cochaperones. In the Tah1-Pih1-CS complex, the Hsp90 "tail" peptide binds in an extended groove lined by the helices forming the TPR domain, and interacts with residues from the first, third, and fifth helices. The conformation of the peptide and its interactions are very similar to those observed for Hsp90 tail peptides bound to the TPR domains of CHIP and AIP (Morgan et al., 2012; Zhang et al., 2005) and essentially identical to those observed in a recent nuclear magnetic resonance analysis of a Tah1-Hsp90 peptide complex (Back et al., 2013) and will not be described

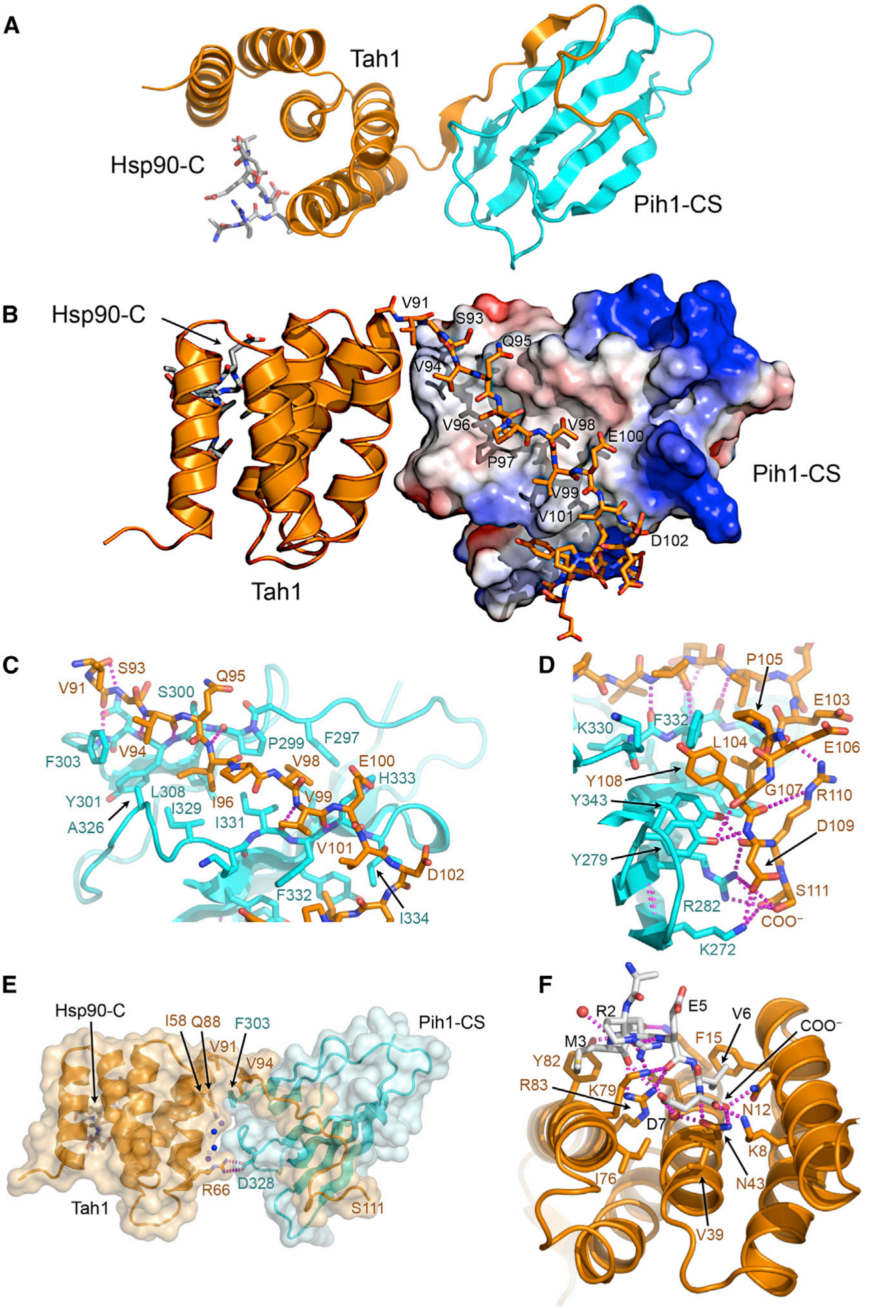

Figure 2. Structure of Tah1- Pih1 Complex

(A) Secondary structure cartoon of TPR domain of Tah1 (gold) bound to the CS domain of Pih1 (cyan) and a peptide derived from the C terminus of Hsp90 (sticks).

(B) Overview of the Tah1-Pih1 complex showing the C-terminal segment of Tah1 (gold, carton and sticks) extending across a shallow hydrophobic depression on the edge of Pih1-CS domain (colored by electrostatic potential: +ve blue → −ve red), and wrapping round to contact the face of the four-stranded β sheet.

(C) The proximal part of the Tah1 C terminus segment bridging the edges of the two β sheets of the Pih1-CS domain β sandwich, with a combination of main chain to main chain hydrogen bonds and hydrophobic interactions.

(D) Interaction of the distal part of the Tah1 C terminus segment, forming a mixed hydrophobic and polar interface with the face of the four-stranded β sheet.

(E) Outside the substantial interaction mediated by the Tah1 C-terminal tail, the juxtaposition of the Tah1-TPR and Pih1-CS domains is fixed by an additional hydrophobic interaction, and an extended polar interface anchored by a bidentate hydrogen bond/ion-pair interaction.

(F) Interactions of the Tah1-TPR domain and Hsp90 C-terminal peptide. The Hsp90 peptide binds with compacted conformation stabilized by interaction of the peptide backbone and side chain of Glu 4, with the side chains of Tah1 residues Lys 50, Lys 79, and Arg 83. The α-carboxyl and carboxylate side chain of Asp 7 is bound by a "carboxylate clamp" formed by Tah1 residues Lys 8, Asn 12, Asn 43, and Lys 79. The side chain of Met 3 in the Hsp90 peptide packs against Tyr 82, but is far more exposed than those on other Hsp90-TPR-domain complexes.

in further detail. The major difference between the Tah1 complex and the CHIP and AIP Hsp90-tail peptide complexes derives from the lack of the two C-terminal α helices in the Tah1 TPR structure. This leaves the methionine residue (M3 in Figure 2F) in the peptide partially exposed, rather than buried in a pocket at the interface of the fifth and seventh helices as in the complexes with CHIP or AIP.

Stoichiometry of the Tah1-Hsp90 Interaction

In metazoa, the role of Tah1 is played by a larger protein, Spagh/RPAP3, which has two tandem TPR domains as well as an uncharacterized N terminus and a C-terminal Monad-binding domain. We therefore used isothermal titration calorimetry (ITC) to investigate whether the stoichiometry for binding of Tah1 and Spagh/RPAP3 to intact Hsp90 differed. Tah1 bound dimeric Hsp90 (K_D = 5.9 μM) with a 1:1 stoichiometry (one dimer Hsp90 and two monomers of Tah1; Figure S1A available online). The affinity (K_D = 4 μM) and stoichiometry were unaffected by the pre-assembled full-length Tah1-Pih1 complex and dimeric Hsp90 (Figures S1A and S1B). The similar affinity and stoichiometry in both cases are consistent with formation of the $(Hsp90)_2$-$(Tah1)_2$ and $(Hsp90)_2$-$(Tah1)_2$-$(Pih1)_2$ complex. Furthermore, a mixture of Tah1, Pih1, and the dimeric C-terminal domain of Hsp90 coeluted from a calibrated gel-filtration chromatography column at a volume fully consistent with formation of a $(Hsp90)_2$-$(Tah1)_2$-$(Pih1)_2$ stoichiometric complex (Figure 3A).

We previously showed that some Hsp90 TPR-domain cochaperones occupy both of the C-terminal MEEVD motifs available in an Hsp90 dimer, preventing the formation of mixed Hsp90-TPR domain cochaperone complexes (Prodromou et al., 1999;

Table 1. Crystallographic Statistics

Data Set	yPih1$^{28–184}$	SeMetTah1 SRMEEVD Pih1$^{187–344}$	Spagh$^{120–255}$ SRMEEVD	Spagh$^{265–380}$ SRMEEVD	SeMet mPih1$^{1–200}$ ELDpSDDEF	mPih1$^{47–179}$ SELDpSDDEF	mPih1$^{47–179}$ SO4
a (Å)	39.75	56.01	52.05	63.57	49.90	50.22	69.86
b (Å)	48.29	78.38	59.12	96.51	61.67	67.73	50.14
c (Å)	96.28	98.79	65.86	52.09	114.26	104.02	35.18
α, β, γ (Å)	90, 90, 90	90, 90, 90	63.42, 67.5, 82.43	90, 90, 90	90, 90, 90	90, 90, 90	90, 103.21, 90
Space group	P21	C 2 2 21	P 1	P 21 21 21	P 21 21 21	P 21 21 21	C 1 2 1
Wavelength (Å)	1.03666	1.5419	0.9200	0.9200	0.9795	0.92	1.54187
Resolution limit (Å)	50–2.03 (2.08–2.03)	39.2–2.11 (2.16–2.11)	54.8–2.54 (2.61–2.54)	48.5–3.0 (3.08–3.0)	54.7–3.06 (3.14–3.06)	56.76–3.3 (3.48–3.3)	34.25–2.1 (2.16-2.1)
Number of observations	23,130 (1,520)	12,533 (623)	20,092 (1,479)	6,373 (469)	68,364 (6,356)	66,348 (5,693)	29,158 (6,845)
Completeness (%)	97.9 (87.6)	97.4 (67.3)	94.0 (94.1)	94.3 (94.6)	90.1 (56.4)	99.6 (99.8)	98.1 (96.4)
Multiplicity	3.0 (2.3)	56.6 (3.0)	2.7 (2.7)	3.3 (3.4)	10.8 (4.3)	11.7 (12.5)	4.3(4.1)
Rmerge (%)	0.071 (0.360)	0.048 (0.210)	0.096 (0.358)	0.080 (0.510)	0.12 (0.519)	0.168 (0.595)	0.076 (0.173)
Rpim(I) (%)	0.061 (0.0354)	0.006 (0.133)	0.082 (0.344)	0.058 (0.362)	0.042 (0.303)	0.056 (0.207)	0.046 (0.176)
Mean I/σI	9.7 (2.2)	83.7 (5.7)	8.1 (2.0)	12.5 (2.0)	15.4 (2.5)	12.8 (4.7)	22.7(14.9)
Refinement							
Resolution Range (Å)	50–2.03	39.2–2.11	54.8–2.6	48.5–3.0	54.7–3.06	56.76–3.3	34.25–2.1
Rcryst	0.1855	0.1688	0.2403	0.2414	0.224	0.2135	0.1278
Rfree	0.2367	0.2101	0.2671	0.2913	0.2814	0.3055	0.1826
No. protein atoms	2,770	1,624	3,923	1,786	2,043	1,778	1,024
No. ligand atoms	12	6	6	0	0	0	21
No. solvent atoms	249	89	80	12	0	15	178
Mean B-factor (Å)	37.13	23.5	43.7	63.2	66.9	16.1	13.7
Rmsd bond lengths (Å)	0.010	0.005	0.005	0.003	0.011	0.01	0.007
Rmsd bond angles (°)	1.04	0.913	0.751	0.671	1.84	1.3	1.02
Ramachandran statistics (%)							
Favored	96.2	98.4	99.2	95	83.9	80.8	98.5
Allowed	3.8	1.6	0.8	5.0	12	17.9	0
Outlier	0	0	0	0	4.1	1.3	1.5

Highest resolution shell in parentheses.

Zhang et al., 2005). To test this with Tah1, we co-immunoprecipitated a dimeric C-terminal construct of Hsp90 with FLAG-tagged Tah1 in the absence or presence of a monomeric TPR-domain cochaperone, Cpr6 (Figure 3B). The levels of C-Hsp90 co-immunoprecipitated by FLAG-Tah1 were progressively decreased as the concentration of Cpr6 present increased, consistent with Cpr6 titrating out the available C-Hsp90. However no Cpr6 was co-immunoprecipitated at any level, indicating that a FLAG-Tah1-$(Hsp90)_2$-Cpr6 mixed cochaperone complex was not formed. This is completely consistent with Tah1 and Hsp90 interacting as a homogeneous 2:2 ($Hsp90_2$:$Tah1_2$) stoichiometric complex, as determined by size exclusion chromatography (Figure 3A), in which both MEEVD sites are occupied.

The absence of the mixed (Tah1 and Cpr6) Hsp90 complex raises the question as to how Tah1 binding excludes simultaneous binding of other monomeric TPR domains. One possibility is that Hsp90-bound Tah1 dimerizes and thus excludes other monomeric TPR domains from binding. Previous work suggested that Tah1 might be able to form dimers, although very weakly, and the biological relevance of this remained undefined (Millson et al., 2008). Using a lysine-specific crosslinker (see Experimental Procedures), we were able to trap a Tah1 dimer species in solution (Figure 3C), supporting the idea that Tah1 may dimerize, a process that would be greatly enhanced once bound to Hsp90, and thus exclude other monomeric TPR domain-containing proteins from forming a mixed TPR domain complex.

Hsp90 Interaction with Spagh/RPAP3

In metazoa, the role of Tah1 is taken by Spagh/RPAP3—a much larger protein with two TPR domains identifiable in its amino acid sequence. We postulated that Spagh/RPAP3 might interact with dimeric Hsp90 as a monomer by utilizing each of its tandem TPR domains to bind to the MEEVD motifs on one Hsp90 dimer. To test this, we analyzed the interaction of the tandem TPR region of human Spagh/RPAP3$^{120–395}$ (TPR_2) with a C-terminal Hsp90 peptide, and with dimeric Hsp90, by ITC at 30°C and 10°C.

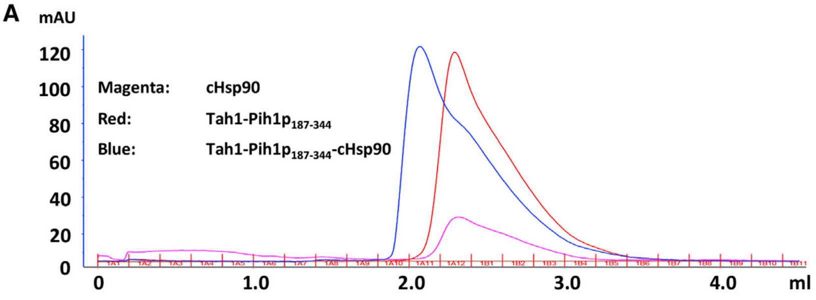

Proteins	Elution volume (ml)	Actual MW (KDa)	Estimated MW (Da)	$Hsp90_2$-$Tah1_2Pih1_2$ Expected MW (Da)
cHsp90	2.32	28	41.7	-
$Tah1$-$Pih1_{187\text{-}344}$	2.3	32	42.6	-
$Tah1$-$Pih1_{187\text{-}344}$-cHsp90	2.08	92	120.2	126.9*

*Expected MW for $Hsp90_2$-Tah_1-$Pih1_1$ is 104 kD

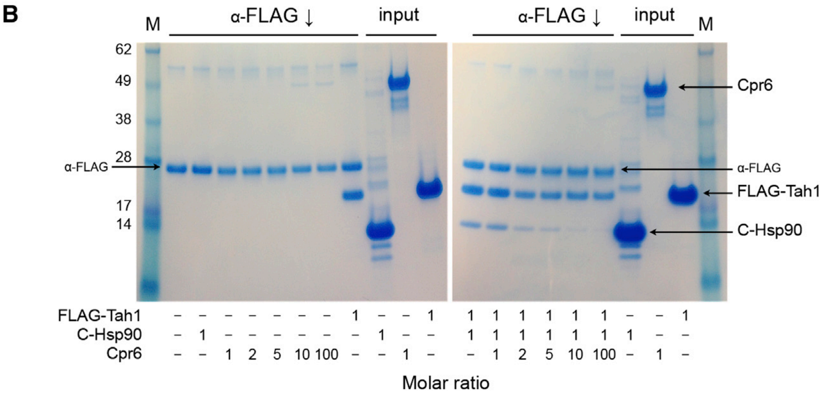

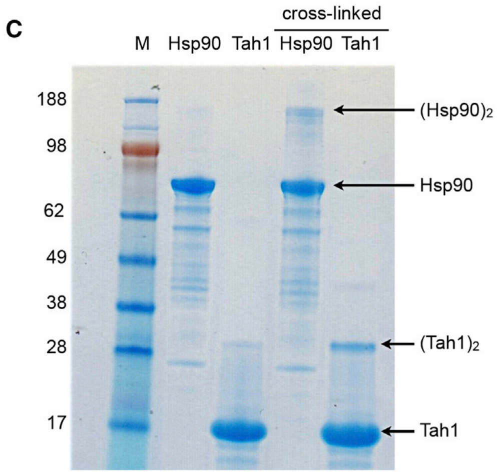

Figure 3. TPR Dimerization and Binding to the Hsp90 Dimer

(A) SEC of cHsp90, Tah1-Pih1p$^{187–344}$, and cHsp90-Tah1-Pih1p$^{187–344}$ complex. The elution volume of the cHsp90-Tah1-Pih1p$^{187–344}$ complex is consistent with a cHsp90$_2$-Tah1$_2$-Pih1p$_2$$^{187–344}$ stoichiometric complex .

(B) Exclusive homogenous occupation of both MEEVD sites on the Hsp90 dimer by Tah1. Left-hand gel: α-FLAG immunoprecipitation controls showing immunoprecipitation of FLAG-Tah1, but no interaction with the TPR-domain Hsp90 cochaperone Cpr6 or the dimeric C-terminal domain of Hsp90. A protein band derived from the α-FLAG antibody system is indicated. Right-hand gel: immunoprecipitation of FLAG-Tah1 coprecipitates Hsp90 C-domain, but the yield is diminished in the presence of increasing concentrations of Cpr6. No Cpr6 is precipitated by FLAG-Tah1 even at very high Cpr6 concentrations, showing that mixed loading of Cpr6 and Tah1 onto the Hsp90 C terminus does not occur and that Tah1 exclusively occupies both MEEVD sites simultaneously.

(C) SDS-PAGE gel showing migration of native Hsp90 and Tah1 proteins and after crosslinking with DMS (see Experimental Procedures). Tah1 shows a substantial yield of crosslinked dimer comparable in level to that obtained for Hsp90, which is a known obligate dimer.

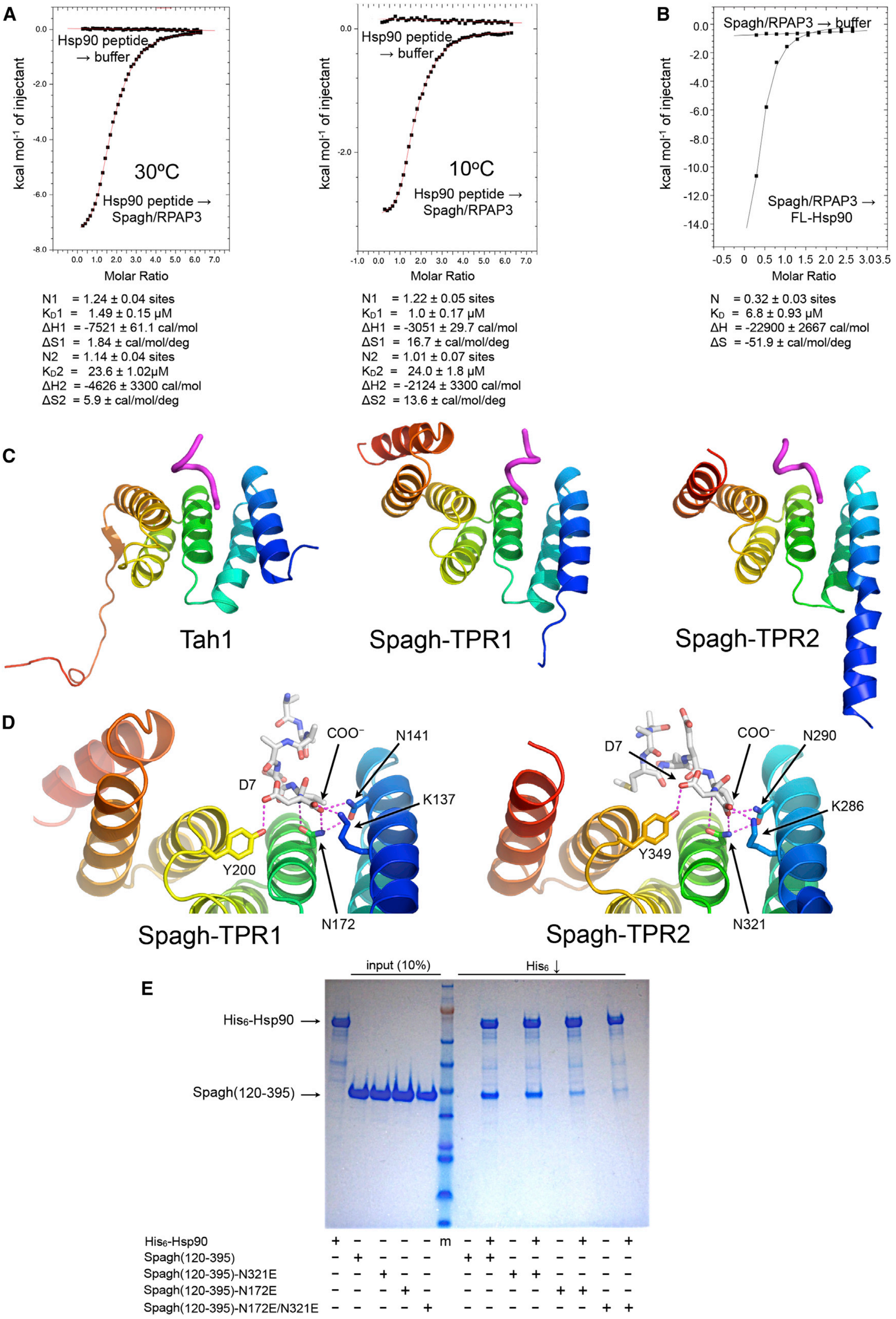

(legend on next page)

Mutant data (see below) suggested a molar ratio indicating two copies of the peptide binding to a single molecule of Spagh/RPAP3-TPR$_2$ and supporting the hypothesis that both Spagh/RPAP3 TPR domains are competent for Hsp90 binding (Figure 4A). Using a two site fitting model, the C-terminal Hsp90 peptide bound to Spagh/RPAP3-TPR$_2$ with tight affinity at one site (K_D = 1.49 and 1.0 μM at 30°C and 10°C, respectively) and with moderate affinity at the other site (K_D = 23.6 and 24.0 μM at 30°C and 10°C, respectively). Consistent with these findings, we found that Spagh/RPAP3-TPR$_2$ bound to a Hsp90 dimer with K_D = 6.8 μM, but with a 1:2 molar ratio, suggesting that a single molecule of Spagh/RPAP3 binds simultaneously to both C-terminal MEEVD motifs of Hsp90 dimer (Figure 4B). The binding of Spagh/RPAP3, and indeed Tah1 and Tah1-Pih1 complex had no major effect on the ATPase activity of yeast Hsp90 and human Hsp90β (Figure S2), consistent with a previous report (Eckert et al., 2010).

We next determined the crystal structures of each of the TPR domains (TPR1 and 2) of human Spagh/RPAP3 cocrystallized with the Hsp90 C-terminal peptide. Spagh-TPR1$^{125–250}$ consists of seven α helices, the first five of which align with the five-helix TPR domain of Tah1. Spagh-TPR2$^{267–381}$ consists of six α helices, with the first five again aligning with Tah1-TPR (Figure 4C). The N-terminal helix of Spagh-TPR2 is double the length of the N-terminal helices of Tah1 or Spagh-TPR1 and projects out of the core TPR domain (Figure 4C). In both Spagh/RPAP3 TPR domains, the Hsp90 C-terminal peptide is bound in the groove formed by the first five α helices. The peptide bound to TPR2 adopts a compacted conformation very similar to the Tah1 complex, making extensive interactions with the walls of the groove. The Hsp90 peptide bound to TPR1 makes fewer interactions and is less well structured, but in both cases the α-carboxyl and the side chain of the C-terminal aspartic acid are bound by a "carboxylate clamp" (Scheufler et al., 2000), anchored by the side chains of Asn 172 (TPR1) and Asn 321 (TPR2), each of which make a bidentate interaction with the peptide NH and α-carboxyl of the C-terminal aspartic acid residue of the Hsp90 peptide (Figure 4D).

We tested the binding model suggested by the ITC experiments by introducing mutations into either Spagh/RPAP3-TPR domain that should disrupt binding of the Hsp90 C-terminal MEEVD motif to that TPR domain only. Mutation of TPR1 (N172E) or TPR2 (N321E) and fitting with a two site model suggested that binding for one site (nonmutant site) remained unaffected (N172E-TPR1 mutant, TPR2: K_D = 9.7 μM and N321E-TPR2 mutant, TPR1: K_D = 0.94 μM), whereas the mutated TPR sites were compromised for binding (N172E-TPR1 mutant, TPR1: K_D = ~231 μM and N321E-TPR2 mutant, TPR2: K_D = ~109 μM; Figures S3A and S3B). The N172E-N321E double mutant failed to show tight binding altogether (Figure S3C), thus confirming that both TPR domains were compromised by the mutations.

Next we used His-tagged Hsp90 in pull-down assays to test whether they bound the TPR domain mutants (Figure 4E) and obtained results that were consistent with those from the ITC experiments. Taken together with the higher affinity and 1:2 molar ratio observed for native Spagh/RPAP3-TPR$_2$ binding to the Hsp90 dimer (one Spagh/RAP3 molecule: one Hsp90 dimer), these data indicate that the two TPR domains bind both C-terminal MEEVD motifs of Hsp90 dimer simultaneously, with the stronger binding TPR1 making the nucleating interaction that facilitates binding of the weaker TPR2.

Structure of Pih1 N-Terminal Domain

Interaction with Tel2 involves the N-terminal segment of Pih1 (Horejsí et al., 2010), which contains a conserved region of unknown structure (henceforth, PIH domain). We determined the crystal structure of the mouse PIH domain$^{1–200}$ (Figure 5A) with single-wavelength anomalous dispersion phasing of Tel2-peptide cocrystals (see below) grown with SeMet protein (see Experimental Procedures) and used this to phase the mPih$^{47–179}$ apo and SELDpSDDEF peptide-bound structures, which were refined to 2.1 and 3.3 Å, respectively (Table 1). We also obtained crystals of an essentially identical region of budding yeast Pih1$^{28–184}$ and determined the structure of this with molecular replacement (Figure 5A).

The PIH domain consists of a twisted five-stranded β sheet with one face traversed by a helix-turn-helix segment connecting strands 4 and 5, and the other face traversed by a coil segment extending from the end of β strand 5. An additional α helix connecting strands 2 and 3 projects from the end of the sheet and packs against the larger of the other two helices.

Pih1-Tel2 Interactions

A number of CK2 phosphorylation sites in a central and probably unstructured segment of Tel2 (Takai et al., 2010) have been implicated in mediating interaction with the R2TP complex (Horejsí et al., 2010). To define which of these are directly involved in the interaction with Pih1, we performed a series of ITC experiments using the mPIH domain and Tel2-derived phosphopeptides.

Figure 4. Structure and Function of Spagh/RPAP3 TPR Domains

(A) ITC binding curve for human Hsp90 C-terminal peptide binding to Spagh/RPAP3 at 30°C (left: K_D1 = 1.49 μM and K_D2 = 23.6 μM, two site fitting) and 10°C (right: K_D1 = 1.0 μM and K_D2 = 24 μM, two site fitting). K_D estimates are consistent between the two temperatures indicating an accurate fit to the data. The peptide binds in an ~2:1 (two Hsp90 peptides to one Spagh/RPAP3) molar ratio consistent with Spagh/RPAP3 mutant binding data (Figures S3A and S3B; TPR1, K_D = 0.94 μM and TPR2, K_D = 9.7 μM).

(B) ITC binding curve for Spagh/RPAP3$^{265–380}$ binding to full-length Hsp90 dimer with an ~1:2 molar ratio with K_D = 6.8 μM.

(C) Structural comparison of yeast Tah1 TPR-domain (left) and TPR1 (center) and TPR2 domains (right) from Spagh/RPAP3. The N-terminal five helices of the Spagh/RPAP3 TPR domains superimpose with the minimal Tah1-TPR domain. The Cα-trace of the bound Hsp90 C-terminal peptide in each case is shown in magenta.

(D) "Carboxyl" clamp interactions with the C terminus of the Hsp90 tail-peptide by Spagh/RPAP3-TPR1 (left) and TPR2 (right). The C-terminal residue of the peptide is anchored on the side chain of Asn 172 in TPR1 and Asn 321 in TPR2.

(E) Pull-down of Spagh/RPAP3$^{120-395}$ tandem TPR segment by His$_6$-Hsp90β. Wild-type Spagh/RPAP3 and a TPR1 mutant (N321E) are efficiently coprecipitated, whereas a TPR2 mutant (N172E) diminishes interaction. Mutation of both TPR domains effectively abolishes coprecipitation with Hsp90, confirming the involvement of both TPRs in Hsp90 interaction.

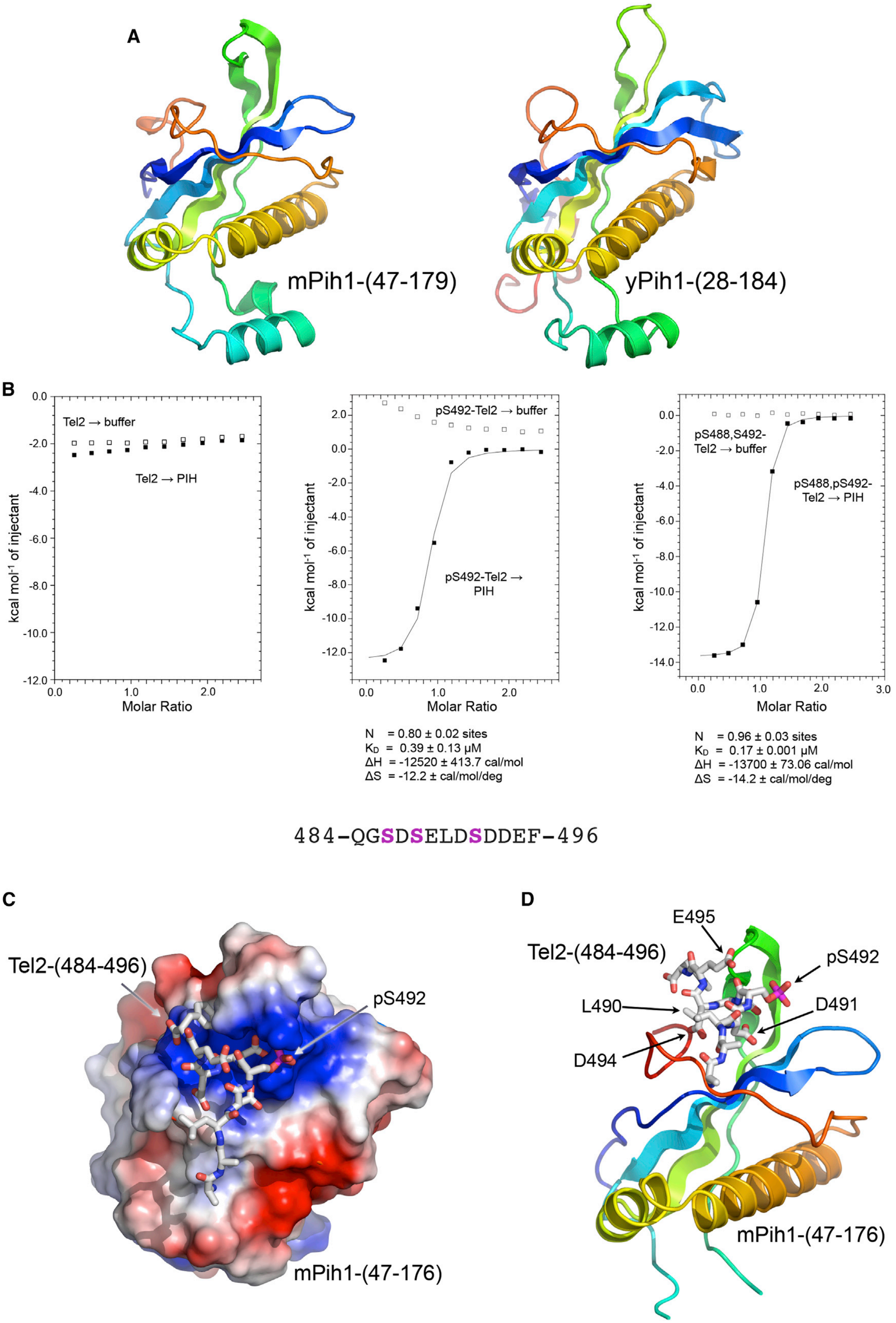

(legend on next page)

Consistent with the requirement for CK2 phosphorylation, an unphosphorylated peptide derived from mouse Tel2 residues 484–496 showed little affinity for the PIH domain (Figure 5B). The same peptide phosphorylated on Ser 486 or Ser 488 alone showed weak to modest binding (K_D > 100 and 15.3 μM, respectively; Figure S4A and SB). In contrast, a pSer 492 and a bis-phosphorylated (pSer 488 and pSer 492) peptide showed significantly higher affinity with K_D = 0.39 and 0.17 μM, respectively (Figure 5B). We also tested the bis-phosphorylated pSer 486 and pSer 488 peptide and found this bound only weakly to the PIH domain (K_D = 37.9 μM; Figure S4C). A Tel2 peptide in which pSer492 was replaced by a phosphothreonine bound substantially weaker than the native phosphoserine version (K_D = 45.7 μM; Figure S4D) suggesting that at least in the context of the Tel2 sequence, the PIH domain is specific for phosphoserine.

We next obtained crystals of a complex of the PIH domain and the Ser 488-Ser 492-bis-phosphorylated Tel2 peptide. The phosphopeptide binds to an intensely basic patch in the high-resolution apo-structure (Figure 5C) formed by the loop connecting β strands 3 and 4; the faces of β strands 1, 2, and 5; and the coil segment C-terminal to β strand 5. Electron density is evident for the Tel2 peptide from residue 488 to 496, but side chains are only well defined for residues 489–495 (Figure 5D).

The Tel2 phosphopeptide binds as a 3_{10} helix stabilized by a backbone peptide hydrogen bond between Asp 491-C=O and Asp 494-NH and reinforced by a hydrogen bond between the side chain of Asp 491 and Asp 493-NH. The core of the PIH domain interaction with the Tel2 phosphopeptide is provided by interlinked hydrogen bonds between the side chains of Lys 57, Lys 64, and Lys 113 from Pih1, and the side chain phosphate and carboxyl groups of pSer 492, Asp 491, and Asp 493 from Tel2. This is reinforced by hydrogen bonds from the side chains of Asp 493 and Asp494 of Tel2 with the peptide NHs of Lys 166 and Ala 112 of Pih1, respectively. An additional hydrophobic interaction is provided by Tel2-Phe 496, which slots into a small crevice formed by the side chains of Ala 112, Arg 168, and Leu 171 of Pih1 (Figure 6A). Tel2-pSer 488 is poorly ordered in the complex and makes no visible interactions with the PIH domain. This is consistent with the weak binding of a Tel2 peptide phosphorylated on this residue alone, and the small additional contribution it makes to the affinity provided by pSer 492, which dominates binding of this segment of Tel2 to the PIH domain.

All the PIH residues involved in interactions with the mouse Tel2 phosphopeptide are conserved in sequence and in the three-dimensional structure in the yeast Pih1 protein, and alignment of mammalian and yeast Tel2 sequences identifies a sequence homologous to the CK2 site in metazoan Tel2 centered on a phosphorylation site at Ser 419 in yeast Tel2 (UniProt P53038). The segment carrying Ser 419 was excised from the polypeptide used in the determination of the yeast Tel2 structure (Takai et al., 2010).

The PIH domain itself and its mode of binding to the Tel2 phosphopeptide are distinct from previously reported phosphoserine/phosphothreonine-binding domains such as BRCT or FHA domains (Ali et al., 2009; Kilkenny et al., 2008; Qu et al., 2013). To verify the binding site in the crystal structure biochemically, we used ITC to measure the binding of the Ser 488-Ser 492-bis-phosphorylated Tel2 peptide to the PIH domain with mutation of residues implicated in phosphopeptide binding by the crystal structure. Consistent with the crystal structure, no or very weak binding was observed with Lys−>Glu charge reversal mutations of Lys 57 or Lys 64 (Figure 6B, left and center). Mutation of the highly conserved Lys 153 on the opposite face of the PIH domain had no effect of Tel2 phosphopeptide binding (Figure 6B, right).

Role of Pih1 Interactions In Vivo

Previous studies revealed the importance of the CK2 sites in Tel2 in connecting the TTT complex to the R2TP/prefoldin and Hsp90 complexes (Horejsí et al., 2010). Consistent with the role of the TTT complex in assembly/stabilization of PIKK proteins (Takai et al., 2010), mutation of these sites resulted in destabilization of Smg1 and mTOR in vivo.

With the detailed structural insights into Pih1 function described here, we sought to confirm the importance of these interactions by mutating Pih1. We constructed budding yeast strains that were (1) lacking Pih1p, (2) expressing a Pih1p truncated at residue 165 and therefore lacking the structurally defined Hsp90-binding CS domain, (3) expressing Pih1p with the biochemically characterized charge reversal mutations that disrupt Tel2 phosphopeptide binding (see above), and (4) Pih1p combining CS domain deletion and Tel2 phosphopeptide-binding site mutations and determined their growth characteristics. The Pih1p-deleted strain showed significant temperature sensitivity relative to wild-type. This temperature sensitivity could be complemented by expression of either the CS-deleted or the Tel2p-binding disrupted Pih1p protein, but not by the Pih1p impaired in interaction with both Hsp90 and Tel2 (Figure 6C). Although the TTT complex has been implicated in stabilization and activation of the DNA damage-sensing PI3K-like kinases ATR and ATM in mammalian systems (Takai et al., 2010), we found no sensitivity to a DNA-damaging agent in the temperature sensitive *pih1Δ* yeast strain relative to wild-type (Figure S5).

Figure 5. Structure of PIH Domain

(A) Secondary structure cartoon of mPih1-PIH domain (left) and yeast Pih1p-PIH domain (right), both rainbow colored. N: blue → C: red.

(B) ITC binding curve for peptides from Tel2 spanning the cluster of putative CK2 phosphorylation sites implicated in mediating interaction with Pih1. The unphosphorylated peptide (left) shows no binding, whereas phosphorylation of Tel2-Ser 492 (center), and bis-phosphorylation of Ser 488 and Ser 492 (right) gives submicromolar affinity with K_D = 0.39 and 0.17 μM, respectively.

(C) Electrostatic surface (blue: +ve, red: −ve) of mPIH domain cocrystal structure with the bis-phosphorylated Tel2 phosphopeptide. The peptide binds to an intensely basic patch complementary to the multiple negative charges carried by the acidic side chains of the phosphorylated peptide. Only residues 486–496 of the bound peptide were visible. The side chain of pSer 486 was only partially visible.

(D) The Tel2-phosphopeptide binds in a compact 3_{10}-helical conformation in a depression formed by the curved face of the central sheet and C-terminal coil segment of the PIH domain. The PIH domain and the mode of binding of the phosphopeptide are quite distinct from previously characterized phosphorylation recognition domains. Only amino acid residues 486–496 of the bound peptide were visible. The side chain of pSer 486 was only partially visible.

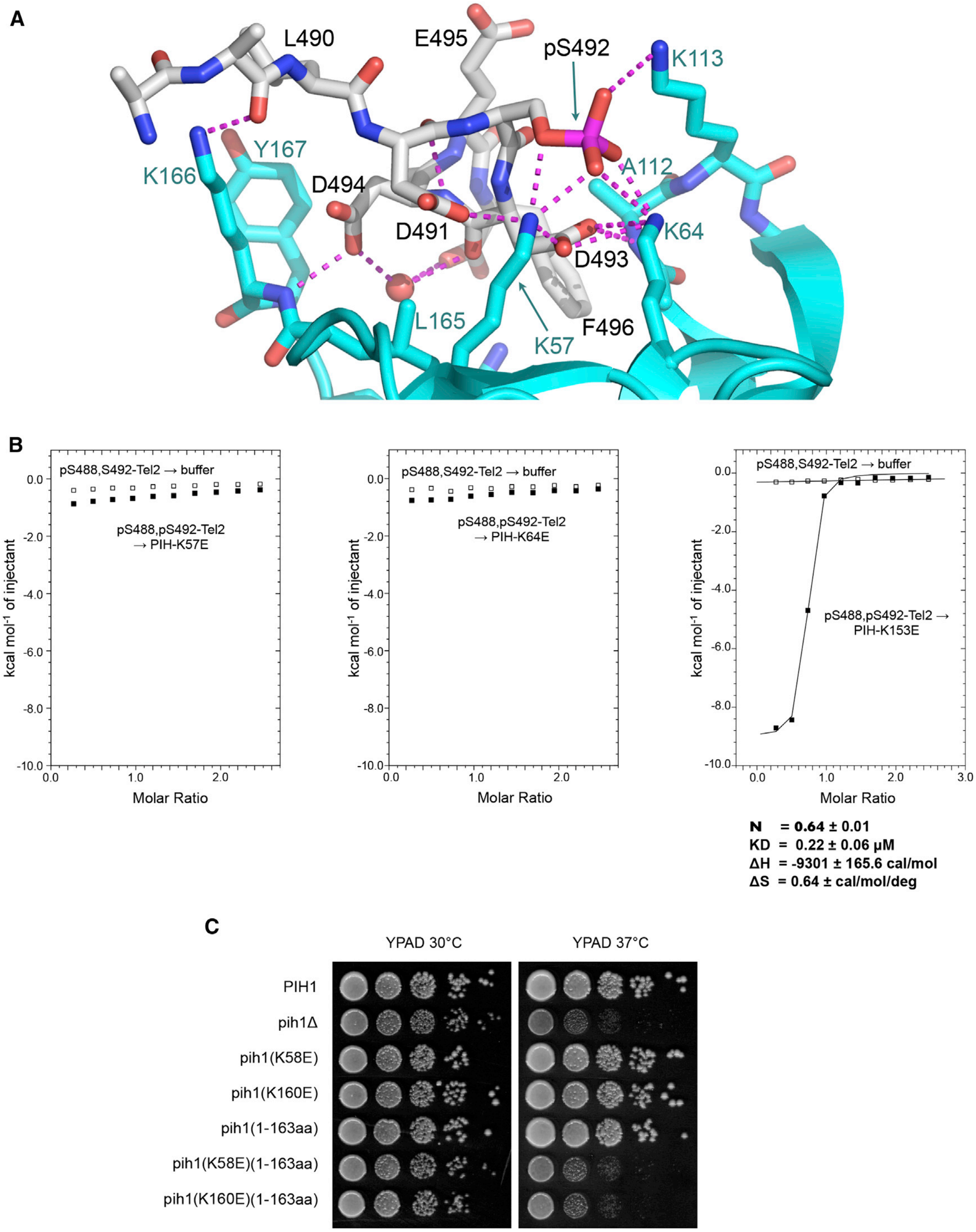

Figure 6. PIH Domain–Tel2 Phosphopeptide Interactions

(A) Interactions between mPIH domain and Tel2 phosphopeptide. The peptide is well ordered from Leu 490 to Phe 496. The core of the interactions is provided by PIH domain residues Lys 57 and Lys 64, which form a network of charge interactions and hydrogen bonds with the carboxyl side chains of Tel2 residues Asp 491 and Asp 493, and the phosphate moiety of pSer 492. Only residues 486–496 of the bound peptide were visible. The side chain of pSer 486 was only partially visible.

(B) Mutation of PIH domain residues Lys 57 (left) or Lys 64 (center) effectively abolishes Tel2-phosphopeptide binding as measured by ITC. Charge reversal of a conserved residue Lys 153, which is not implicated in Tel2 binding by the crystal structure, has no effect on the affinity of the PIH domain for the phosphopeptide (right).

(C) Growth of yeast with modified Pih1. Deletion of Pih1 shows a temperature-sensitive (ts) phenotype, whereas deletion of only the CS domain (Pih1$^{1-163}$), which mediates recruitment to Hsp90, is not ts. Mutation of residues implicated in phospho-Tel2 binding (K58 equivalent to mouse K64, and K160 equivalent to mouse K166) in isolation did not generate a ts phenotype; however, simultaneous disruption of Tel2 binding and Hsp90 recruitment elicits the ts growth phenotype.

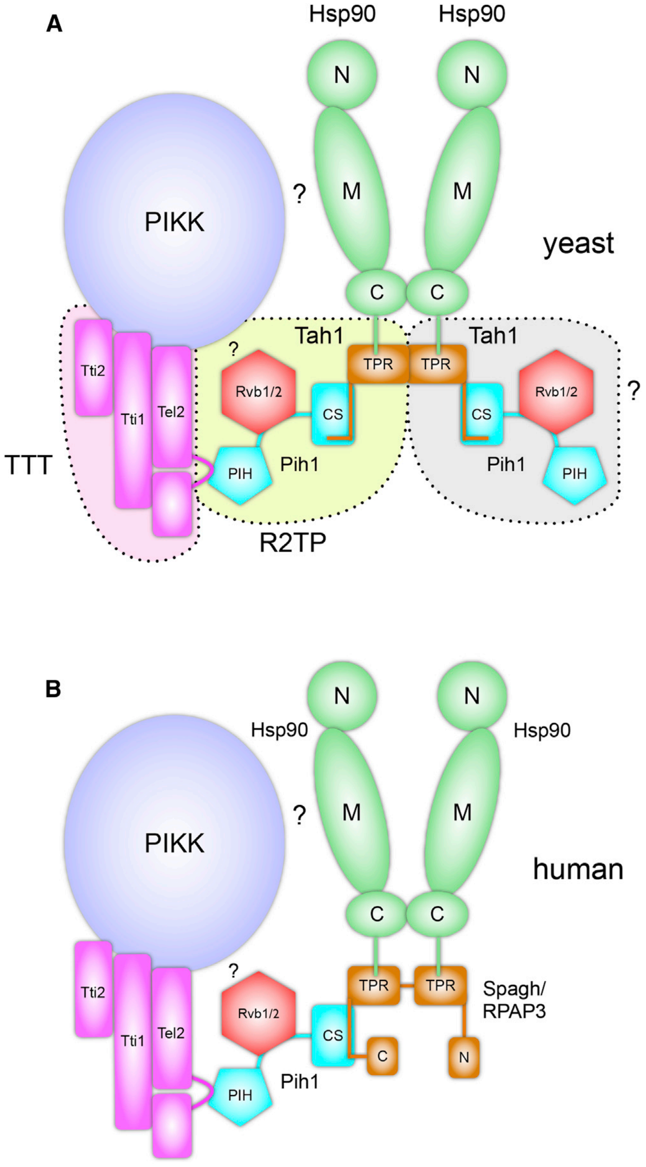

Figure 7. Assembly of the Hsp90-R2TP-TTT Supercomplex

(A) Structure of the overall yeast Hsp90-R2TP-TTT supercomplex required for activation of PIKK enzymes such as TOR. The data presented here provide detailed insights into the chain of protein-protein interactions connecting the C terminus of Hsp90 to the TPR domain of Tah1, the C-terminal segment of Tah1 to the CS-domain of Pih1, and the PIH-domain of Pih1 to the CK2 sites on Tel2. Whether Rvb1/2 and Hsp90 directly contact the client PIKK remains to be determined. Tah1 binds Hsp90 as a dimer, occupying both TPR-binding sites on an Hsp90 dimer. However, whether this causes recruitment of a second Pih1-complex and potentially an additional TTT-PIKK complex is unknown.

(B) As in (A), but for the metazoan system where Tah1 function is replaced by Spagh/RPAP3, which binds both TPR-binding sites on an Hsp90 dimer as a single polypeptide. The role of the additional N- and C-terminal domains of Spagh/RPAP3 in PIKK activation remains to be determined.

DISCUSSION

The Hsp90 system is implicated in assembly, stabilization, and activation of a plethora of proteins and complexes including protein kinases (conventional and PIKK), steroid hormone receptors, NLR innate immunity receptors, and both viral and cellular DNA and RNA polymerases (Pearl et al., 2008). Recruitment of this wide-ranging clientele to Hsp90 is mediated by cochaperones, adaptors that interact simultaneously with Hsp90 and the specific client protein class. Conventional protein kinases, for example, are recruited by Cdc37 (Pearl, 2005), which arrests the ATPase-coupled conformational cycle of Hsp90 (Siligardi et al., 2002) and silences the kinase activity of the client (Polier et al., 2013).

PIKKs are recruited by a far more complicated system involving a chain of protein connections, mediated by an Hsp90-binding TPR-domain protein (Tah1 or Spagh/RPAP3) coupled to the CS-domain of Pih1. The PIH-domain of Pih1 then binds the Tel2 component of the TTT complex, which in turn binds the client (Kakihara and Houry, 2012). Pih1 also binds the AAA-ATPases Rvb1 and Rvb2 via a segment between the PIH and CS domains (Paci et al., 2012; Figures 7A and 7B). The mechanistic role of the Rvb1/Rvb2 complex, which is implicated in numerous processes as well as PIKK stabilization/activation, is unknown. However, it probably involves Pih1-mediated interaction of Rvb1/Rvb2 with the PIKK client and may be partly redundant with Hsp90 function because disruption of both Tah1-binding (and hence Hsp90 association) by Pih1, and phospho-specific interaction of Pih1 with Tel2, were required to phenocopy Pih1 deletion in yeast cells.

The R2TP-TTT nexus is a more protracted and convoluted link between client and chaperone than for the conventional protein kinases, which form an intimate complex with Hsp90 and Cdc37 (Vaughan et al., 2006), and it is not clear whether, and if so, how, Hsp90 and a PIKK client protein interact directly. Nonetheless, as with conventional protein kinases, the ATPase activity of the chaperone plays an important role and its pharmacological inhibition impairs PIKK stability (Takai et al., 2010).

Spagh/RPAP3 and Tah1 Interaction with Hsp90

The TPR1 and TPR2 domains of Spagh/RPAP3 have a typical TPR domain structure, but lack the distinct hydrophobic pocket formed between helices 5 and 7, into which the methionine side chain of the MEEVD motif binds (Morgan et al., 2012; Zhang et al., 2005). Instead and similar to Tah1, the methionine side chain packs against the surface of helix 5 and remains partially exposed. Whether this mode of binding, which is conserved between Tah1 and Spagh/RPAP3, has any special biological significance remains to be determined.

Although they fulfil the same role of bridging Pih1 to Hsp90, Tah1 and Spagh/RPAP3 have significant structural and functional differences. Our data show that two molecules of Tah1 can bind to an Hsp90 dimer and that each of these can recruit a molecule of Pih1, generating a fully symmetric $(Hsp90)_2$-$(Tah1)_2$-$(Pih1)_2$ complex. In principle, this dimer symmetry may be further propagated into complexes with TTT and other factors that Pih1 mediates, but whether this actually occurs in vivo remains to be determined. In contrast we find that only a single molecule of Spagh/RPAP3 binds to an Hsp90 dimer, generating an inherently asymmetric $(Hsp90)_2$-$(Spagh/RPAP3)_1$ complex capable of recruiting a single Pih1 and downstream partners (Figure 7).

PIH domain Is a Phosphopeptide-Binding Module

Our structural and mutational data characterize the PIH domain as a phosphopeptide-binding domain that appears exquisitely adapted to providing specific interactions with phosphoserines (and possibly phosphothreonines) embedded in highly acidic surrounding sequences, characteristic of CK2 phosphorylation sites. The interaction of the PIH domain and the Tel2 phosphopeptide is highly unusual when compared with other known phosphopeptide binding domains. The phosphoserine phosphate group and the side chain carboxyls of the flanking aspartic acid residues on the Tel2 peptide cooperate in an interconnecting network of polar interactions with lysine residues 57 and 64 in the PIH domain. An additional interaction with the PIH domain main chain is provided by the aspartic acid side chain +2 relative to the phosphoserine. Whereas our data show that the PIH domain has an absolute requirement for a phosphoserine for binding, the surrounding acidic residues also make a substantial contribution, so that a Tel2-pSer 486 peptide, which lacks the −1 and +2 aspartic acid residues, only binds very weakly, while the Tel2-pSer 488 peptide, which has acidic residues at −1 and +1, binds with ~15 μM affinity, but still ~40-fold weaker than the Tel2-pSer 492 peptide with the full DpSDD core sequence. An additional source of specificity may be contributed by the hydrophobic interaction of Tel2-Phe 496, positioned +4 relative to the phosphoserine. Tel2 sequences from a range of metazoa conserve a large hydrophobic residue at this position, suggesting a functional role.

Because Hsp90 and the R2TP complex are individually and collaboratively implicated in a range of biological processes, we speculated that the PIH domain of Pih1 might facilitate phosphorylation-dependent recruitment of other proteins in addition to Tel2. To test this, we performed a bioinformatics search of known phosphorylation sites curated in PhosphoSitePlus (Hornbeck et al., 2004) using a search motif based on the high-affinity Tel2-p492 sequence D[pSpT]DDx[FLIM]. Although replacement of phosphoserine by phosphothreonine in the Tel2 motif diminished the affinity for Pih1 (Figure S4D), we considered the possibility that this effect might be context specific, and so we allowed for either phosphorylated residue in the search motif. The search yielded 46 putative binding sites in human proteins (Table S1), although none in known components of R2TP-dependent systems such as snoRNPs or RNA polymerases. The identified proteins function in a wide range of biological processes and whether their matching phosphorylation sites mediate biologically significant interactions remains to be seen. Within these, the search motif identifies a well-documented and conserved phosphorylation site (554-MANDpSDDSIS-563) in the DNA double-strand break recognition and resection protein, Mre11. A phosphopeptide based on this sequence (MANDpSDDSI) bound the Pih1-PIH domain with low micromolar affinity (K_D = 6.4 μM; Figure S6) in ITC experiments, suggesting that the interaction could occur in vivo. ATM interaction and focus formation by the MRN (Mre11-Rad50-Nbs1) complex is impaired in cells treated with Hsp90 inhibitors, and the MRN complex and Hsp90 coprecipitate (Dote et al., 2006), but the nature of the presumed cochaperone system that mediates Hsp90-MRN interaction has not been determined. The presence of a functional PIH domain-binding motif in Mre11 raises the intriguing possibility that Hsp90 recruitment is mediated by R2TP, but further work will be required to test this.

A domain homologous to the PIH domain structurally characterized here has been identified in kintoun/DNAAF2/PF13, a factor required for cytoplasmic assembly of dynein arm complexes prior to their transport into cilia (Omran et al., 2008; Yamamoto et al., 2010). Residues in the mPIH domain involved in interacting with the phosphorylated Tel2 CK2 site are conserved in kintoun/DNAAF2/PF13, suggesting that it also mediates phosphorylation-dependent protein-protein interactions, although the target of this is currently unknown. A CS domain homologous to that in the C-terminal region of Pih1 is also detectable in kintoun/DNAAF2/PF13 immediately C-terminal of the PIH domain, with a second CS domain identifiable downstream. Kintoun/DNAAF2/PF13 has recently been shown to interact with DYX1C1 (Tarkar et al., 2013), a TPR-domain and CS-domain protein also implicated in axonemal dynein assembly and mutated in human primary ciliary dyskinesia. The DYX1C1 TPR domain conserves the pattern of residues involved in binding the Hsp90 and/or Hsp70 C-terminal EEVD motif (see above), and these chaperones are enriched in DYX1C1 cellular coprecipitates. While the details of these interactions are yet to be fully described, it is likely that the chain of TPR, CS, and PIH domains that mediate Hsp90 involvement with PIKKs via the R2TP system structurally characterized here also play a key role in connecting Hsp90 to the dynein assembly.

EXPERIMENTAL PROCEDURES

Protein Purification

pTwo-E expressed His-mPih1$^{1–200}$, mutants and mPIH1$^{47–179}$, were purified by Talon affinity, gel filtration, and ion-exchange chromatography. mPih1 constructs were purified by Talon affinity and gel filtration chromatography. Tah1p and the Tah1p-Pih1p$^{187–344}$ complex was expressed in *Escherichia coli* Rosetta (DE3) pLysS cells. Tah1p and the Tah1p-Pih1p$^{187–344}$ complex were purified by Talon affinity and gel filtration chromatography. Spagh/RPAP3$^{120–395}$ was expressed as a GST PreScission fusion and purified by GST affinity and gel filtration chromatography. For selenomethionine (SeMet) protein labeling, cells were grown in media containing SeMet.

Crystallography

Crystallizations were conducted at 10 mg/ml. The Tah1p-Pih1p$^{187–344}$-SRMEEVD complex was crystallized in 25% PEG 1500 (w/v) and a 100 mM MIB buffer pH 6.0 (a mixture of malonic acid, imidazole, and boric acid) at 4°C. Crystals for TPR1 of RPAP3 were obtained in 100 mM HEPES, pH 7.0, and 20% PEG 8000 (w/v) at 4°C, whereas those for TPR2 in were obtained in 0.1 M Na-citrate pH 5.6, 20% PEG 4000 (w/v), and 20% 2-propanol at 4°C. Crystals of TPR1 and TPR2 were obtained following proteolysis of Spagh/RPAP3$^{200–395}$ during crystallization. SeMet-mPih1$^{1–200}$ -Tel2 phosphopeptide (QGSDpSELDpSDDEF) was crystallized at 14°C in 0.1 M PCTP buffer pH 8.0, 25% PEG 1500 (w/v). In contrast, mPih1$^{47–179}$-Tel2 phosphopeptide was crystallized at 14°C in 0.15 M $NaKHPO_4$, 20% PEG 3350, and 0.1 M Bis-Tris pH 6.5. Apo mPih1$^{47–179}$ crystallized in 0.02 M $MgCl_2.6H_20$, 0.002M $CoCl_2$, 0.05M HEPES pH 7.5, 2M $(NH_4)_2SO_4$, and 0.001 M Spermine.

Crystals were harvested with glycerol (mPih1 constructs) or ethylene glycol (all other constructs) and flash-frozen. Diffraction data were collected at 100 K either on a Rigaku 007HF generator (λ = 1.5419 Å) or at Diamond Light Source UK. Data were processed with CCP4 (Krissinel et al., 2004) and Phenix (Afonine et al., 2012), and manual rebuilding was performed in Coot (Emsley and Cowtan, 2004). The Spagh/RPAP3-TPR1- and TPR2-peptide complexes were solved by molecular replacement with the Tah1p structure. Data for apo mPih1$^{47–179}$, mPih1$^{47–179}$-Tel2, and SeMet-mPih1$^{1–200}$-Tel2 complexes were integrated, and scaled with XDS (Kabsch, 2010), and graphics were depicted with PyMol (Schrödinger).

Isothermal Titration Calorimetry and ATPase Assays

Heat of interaction was measured on an ITC_{200} microcalorimeter (Microcal) under the same buffer conditions (20 mM Tris, pH 7.5, containing 5 mM NaCl). Aliquots of the Tel2 peptides at 350 μM were injected into 30 μM of Pih1 at 30°C. Interactions with Tah1 or Spagh/RPAP3$^{120–395}$ were performed in 10 mM Tris pH 7.5 and 5 mM NaCl at 10°C and 30°C. Aliquots of 600 μM Tah1 were injected into 40 μM of yeast Hsp90, 400 μM of SRMEEVD injected into 30 μM Tah1, 301 μM yeast Hsp90 injected into 25 μM yeast Tah1-Pih1 complex, and 400 μM SRMEEVD injected into 30 μM yeast Tah1-Pih1 complex. For Spagh/RPAP3$^{120–395}$ experiments, aliquots of 500 μM yeast Hsp90 were injected into 20 μM Spagh/RPAP3$^{120–395}$ or 2 mM DDTSRMEEVD into 80 μM Spagh/RPAP3$^{120–395}$ or mutants (N172E, N321E, and N172E-N321E). Heats of dilution were determined by diluting injectant into buffer. Data were fitted using a curve-fitting algorithm (Microcal Origin). ATPase assays were previously described (Prodromou et al., 1999).

Cross-Linking and Pull-Downs

Increasing concentrations of Cpr6 and 70 μM Flag-Tah1p, Hsp90$^{600–709}$ were incubated in 20 mM HEPES, pH 7.5, 150 mM NaCl, 10% glycerol, 0.5% Igepal, and 2 mM EDTA. Subsequently, 30 μl Anti-Flag M2 magnetic beads (Sigma-Aldrich) were added. Beads were then washed, and the elute was depicted with SDS-PAGE. Cross-linking experiments were previously described (Prodromou et al., 2000).

Yeast Strains and Plasmids

The *pih1* deletion strain (*pih1*::KanR in BY4741) was obtained from Euroscarf. The *Pih1* promoter and the full coding sequence were amplified cloned into the *Not*I site of pRS415 (Stratagene). Truncated *Pih1* was constructed by inserting a stop codon at Leu 168 and cloned into the *Not*I site of pRS415. The K58E or K106E mutations were introduced into either the full-length or truncated *Pih1* construct by site-directed mutagenesis.

Survival and Growth Assays

Yeast transformed with Pih1 expression constructs were grown in synthetic complete media lacking leucine (SC-His). To determine temperature sensitivity, 5-fold dilutions of logarithmically growing cultures were spotted onto plates and incubated at the permissive (30°C) or nonpermissive temperature (37°C) and imaged after 3 days. For growth rate analysis, yeast strains grown to saturation in SC-His were diluted into fresh media and the optical density at 600 nm was measured. Data are represented as the average of three independent experiments ± 1 SD.

ACCESSION NUMBERS

The Protein Data Bank accession numbers for the coordinates and structure factors are SeMetTah1-SRMEEVD-Pih1$^{187–344}$, 4CGU; RPAP3/Spagh$^{120-255}$-SRMEEVD, 4CGV; RPA3/Spagh$^{265–380}$-SRMEEVD, 4CGW; yeastPih1, 4CHH; SeMet mPih1$^{1–200}$-ELDpSDDEF, 4CKT; mPih1$^{47–179}$-SELDpSDDEF, 4CSE; and mPih1$^{47–179}$ -SO_4, 4CV4.

SUPPLEMENTAL INFORMATION

Supplemental Information includes six figures and one table and can be found with this article online at http://dx.doi.org/10.1016/j.str.2014.04.001.

AUTHOR CONTRIBUTIONS

M.P., M.M., and C.P. prepared expression systems, purified and crystallized proteins, and performed in vitro assays; S.E.L.P. and J.A.D. performed in vivo yeast experiments; S.P.-M. and S.P. prepared expression systems; S.M.R., M.M., and M.P. determined crystal structures; and L.H.P. and C.P. designed the study, wrote the manuscript and prepared the figures.

ACKNOWLEDGMENTS

We are grateful to the Diamond Light Source Ltd., Didcot, UK, for access to synchrotron radiation and to the Wellcome Trust for support for X-ray diffraction facilities at the University of Sussex. This work was supported by Wellcome Trust Senior Investigator award 095605/Z11/Z (to L.H.P.) and Cancer Research UK Career Enhancement award C7905/A10269 (to J.A.D.). L.H.P. acknowledges funding for the Genome Damage and Stability Centre from the Medical Research Council. S.E.L.P. is supported by a University of Sussex PhD studentship.

Received: January 26, 2014
Revised: March 24, 2014
Accepted: April 4, 2014
Published: May 1, 2014

REFERENCES

Afonine, P.V., Grosse-Kunstleve, R.W., Echols, N., Headd, J.J., Moriarty, N.W., Mustyakimov, M., Terwilliger, T.C., Urzhumtsev, A., Zwart, P.H., and Adams, P.D. (2012). Towards automated crystallographic structure refinement with phenix.refine. Acta Crystallogr. D Biol. Crystallogr. *68*, 352–367.

Ali, M.M., Roe, S.M., Vaughan, C.K., Meyer, P., Panaretou, B., Piper, P.W., Prodromou, C., and Pearl, L.H. (2006). Crystal structure of an Hsp90-nucleotide-p23/Sba1 closed chaperone complex. Nature *440*, 1013–1017.

Ali, A.A., Jukes, R.M., Pearl, L.H., and Oliver, A.W. (2009). Specific recognition of a multiply phosphorylated motif in the DNA repair scaffold XRCC1 by the FHA domain of human PNK. Nucleic Acids Res. *37*, 1701–1712.

Back, R., Dominguez, C., Rothé, B., Bobo, C., Beaufils, C., Moréra, S., Meyer, P., Charpentier, B., Branlant, C., Allain, F.H., and Manival, X. (2013). High-resolution structural analysis shows how Tah1 tethers Hsp90 to the R2TP complex. Structure *21*, 1834–1847.

Boulon, S., Pradet-Balade, B., Verheggen, C., Molle, D., Boireau, S., Georgieva, M., Azzag, K., Robert, M.C., Ahmad, Y., Neel, H., et al. (2010). HSP90 and its R2TP/Prefoldin-like cochaperone are involved in the cytoplasmic assembly of RNA polymerase II. Mol. Cell *39*, 912–924.

Dote, H., Burgan, W.E., Camphausen, K., and Tofilon, P.J. (2006). Inhibition of hsp90 compromises the DNA damage response to radiation. Cancer Res. *66*, 9211–9220.

Eckert, K., Saliou, J.M., Monlezun, L., Vigouroux, A., Atmane, N., Caillat, C., Quevillon-Chéruel, S., Madiona, K., Nicaise, M., Lazereg, S., et al. (2010). The Pih1-Tah1 cochaperone complex inhibits Hsp90 molecular chaperone ATPase activity. J. Biol. Chem. *285*, 31304–31312.

Emsley, P., and Cowtan, K. (2004). Coot: model-building tools for molecular graphics. Acta Crystallogr. D Biol. Crystallogr. *60*, 2126–2132.

Horejsí, Z., Takai, H., Adelman, C.A., Collis, S.J., Flynn, H., Maslen, S., Skehel, J.M., de Lange, T., and Boulton, S.J. (2010). CK2 phospho-dependent binding of R2TP complex to TEL2 is essential for mTOR and SMG1 stability. Mol. Cell *39*, 839–850.

Hornbeck, P.V., Chabra, I., Kornhauser, J.M., Skrzypek, E., and Zhang, B. (2004). PhosphoSite: A bioinformatics resource dedicated to physiological protein phosphorylation. Proteomics *4*, 1551–1561.

Hurov, K.E., Cotta-Ramusino, C., and Elledge, S.J. (2010). A genetic screen identifies the Triple T complex required for DNA damage signaling and ATM and ATR stability. Genes Dev. *24*, 1939–1950.

Izumi, N., Yamashita, A., Hirano, H., and Ohno, S. (2012). Heat shock protein 90 regulates phosphatidylinositol 3-kinase-related protein kinase family proteins together with the RUVBL1/2 and Tel2-containing co-factor complex. Cancer Sci. *103*, 50–57.

Jiménez, B., Ugwu, F., Zhao, R., Ortí, L., Makhnevych, T., Pineda-Lucena, A., and Houry, W.A. (2012). Structure of minimal tetratricopeptide repeat domain protein Tah1 reveals mechanism of its interaction with Pih1 and Hsp90. J. Biol. Chem. *287*, 5698–5709.

Kabsch, W. (2010). Xds. Acta Crystallogr. D Biol. Crystallogr. *66*, 125–132.

Kakihara, Y., and Houry, W.A. (2012). The R2TP complex: discovery and functions. Biochim. Biophys. Acta *1823*, 101–107.

Kilkenny, M.L., Doré, A.S., Roe, S.M., Nestoras, K., Ho, J.C., Watts, F.Z., and Pearl, L.H. (2008). Structural and functional analysis of the Crb2-BRCT2 domain reveals distinct roles in checkpoint signaling and DNA damage repair. Genes Dev. *22*, 2034–2047.

Krissinel, E., and Henrick, K. (2007). Inference of macromolecular assemblies from crystalline state. J. Mol. Biol. *372*, 774–797.

Krissinel, E.B., Winn, M.D., Ballard, C.C., Ashton, A.W., Patel, P., Potterton, E.A., McNicholas, S.J., Cowtan, K.D., and Emsley, P. (2004). The new CCP4 Coordinate Library as a toolkit for the design of coordinate-related applications in protein crystallography. Acta Crystallogr. D Biol. Crystallogr. *60*, 2250–2255.

Millson, S.H., Vaughan, C.K., Zhai, C., Ali, M.M., Panaretou, B., Piper, P.W., Pearl, L.H., and Prodromou, C. (2008). Chaperone ligand-discrimination by the TPR-domain protein Tah1. Biochem. J. *413*, 261–268.

Morgan, R.M., Hernández-Ramírez, L.C., Trivellin, G., Zhou, L., Roe, S.M., Korbonits, M., and Prodromou, C. (2012). Structure of the TPR domain of AIP: lack of client protein interaction with the C-terminal α-7 helix of the TPR domain of AIP is sufficient for pituitary adenoma predisposition. PLoS ONE *7*, e53339.

Omran, H., Kobayashi, D., Olbrich, H., Tsukahara, T., Loges, N.T., Hagiwara, H., Zhang, Q., Leblond, G., O'Toole, E., Hara, C., et al. (2008). Ktu/PF13 is required for cytoplasmic pre-assembly of axonemal dyneins. Nature *456*, 611–616.

Paci, A., Liu, X.H., Huang, H., Lim, A., Houry, W.A., and Zhao, R. (2012). The stability of the small nucleolar ribonucleoprotein (snoRNP) assembly protein Pih1 in Saccharomyces cerevisiae is modulated by its C terminus. J. Biol. Chem. *287*, 43205–43214.

Pearl, L.H. (2005). Hsp90 and Cdc37 — a chaperone cancer conspiracy. Curr. Opin. Genet. Dev. *15*, 55–61.

Pearl, L.H., Prodromou, C., and Workman, P. (2008). The Hsp90 molecular chaperone: an open and shut case for treatment. Biochem. J. *410*, 439–453.

Polier, S., Samant, R.S., Clarke, P.A., Workman, P., Prodromou, C., and Pearl, L.H. (2013). ATP-competitive inhibitors block protein kinase recruitment to the Hsp90-Cdc37 system. Nat. Chem. Biol. *9*, 307–312.

Prodromou, C., Siligardi, G., O'Brien, R., Woolfson, D.N., Regan, L., Panaretou, B., Ladbury, J.E., Piper, P.W., and Pearl, L.H. (1999). Regulation of Hsp90 ATPase activity by tetratricopeptide repeat (TPR)-domain co-chaperones. EMBO J. *18*, 754–762.

Prodromou, C., Panaretou, B., Chohan, S., Siligardi, G., O'Brien, R., Ladbury, J.E., Roe, S.M., Piper, P.W., and Pearl, L.H. (2000). The ATPase cycle of Hsp90 drives a molecular 'clamp' via transient dimerization of the N-terminal domains. EMBO J. *19*, 4383–4392.

Qu, M., Rappas, M., Wardlaw, C.P., Garcia, V., Ren, J.Y., Day, M., Carr, A.M., Oliver, A.W., Du, L.L., and Pearl, L.H. (2013). Phosphorylation-dependent assembly and coordination of the DNA damage checkpoint apparatus by Rad4(TopBP^1). Mol. Cell *51*, 723–736.

Scheufler, C., Brinker, A., Bourenkov, G., Pegoraro, S., Moroder, L., Bartunik, H., Hartl, F.U., and Moarefi, I. (2000). Structure of TPR domain-peptide complexes: critical elements in the assembly of the Hsp70-Hsp90 multichaperone machine. Cell *101*, 199–210.

Siligardi, G., Panaretou, B., Meyer, P., Singh, S., Woolfson, D.N., Piper, P.W., Pearl, L.H., and Prodromou, C. (2002). Regulation of Hsp90 ATPase activity by the co-chaperone Cdc37p/p50^{cdc37}. J. Biol. Chem. *277*, 20151–20159.

Takai, H., Xie, Y., de Lange, T., and Pavletich, N.P. (2010). Tel2 structure and function in the Hsp90-dependent maturation of mTOR and ATR complexes. Genes Dev. *24*, 2019–2030.

Tarkar, A., Loges, N.T., Slagle, C.E., Francis, R., Dougherty, G.W., Tamayo, J.V., Shook, B., Cantino, M., Schwartz, D., Jahnke, C., et al.; UK10K (2013). DYX1C1 is required for axonemal dynein assembly and ciliary motility. Nat. Genet. *45*, 995–1003.

Te, J., Jia, L., Rogers, J., Miller, A., and Hartson, S.D. (2007). Novel subunits of the mammalian Hsp90 signal transduction chaperone. J. Proteome Res. *6*, 1963–1973.

Vaughan, C.K., Gohlke, U., Sobott, F., Good, V.M., Ali, M.M., Prodromou, C., Robinson, C.V., Saibil, H.R., and Pearl, L.H. (2006). Structure of an Hsp90-Cdc37-Cdk4 complex. Mol. Cell *23*, 697–707.

Yamamoto, R., Hirono, M., and Kamiya, R. (2010). Discrete PIH proteins function in the cytoplasmic preassembly of different subsets of axonemal dyneins. J. Cell Biol. *190*, 65–71.

Zhang, M., Windheim, M., Roe, S.M., Peggie, M., Cohen, P., Prodromou, C., and Pearl, L.H. (2005). Chaperoned ubiquitylation—crystal structures of the CHIP U box E3 ubiquitin ligase and a CHIP-Ubc13-Uev1a complex. Mol. Cell *20*, 525–538.

Zhang, M., Botër, M., Li, K., Kadota, Y., Panaretou, B., Prodromou, C., Shirasu, K., and Pearl, L.H. (2008). Structural and functional coupling of Hsp90- and Sgt1-centred multi-protein complexes. EMBO J. *27*, 2789–2798.

Zhao, R., Davey, M., Hsu, Y.C., Kaplanek, P., Tong, A., Parsons, A.B., Krogan, N., Cagney, G., Mai, D., Greenblatt, J., et al. (2005). Navigating the chaperone network: an integrative map of physical and genetic interactions mediated by the hsp90 chaperone. Cell *120*, 715–727.

Structure, Volume *22*

Supplemental Information

Structural Basis for Phosphorylation-Dependent Recruitment of Tel2 to Hsp90 by Pih1

Mohinder Pal, Marc Morgan, Sarah E.L. Phelps, S. Mark Roe, Sarah Parry-Morris, Jessica A. Downs, Sigrun Polier, Laurence H. Pearl, and Chrisostomos Prodromou

Figure S1 – related to Figure 2

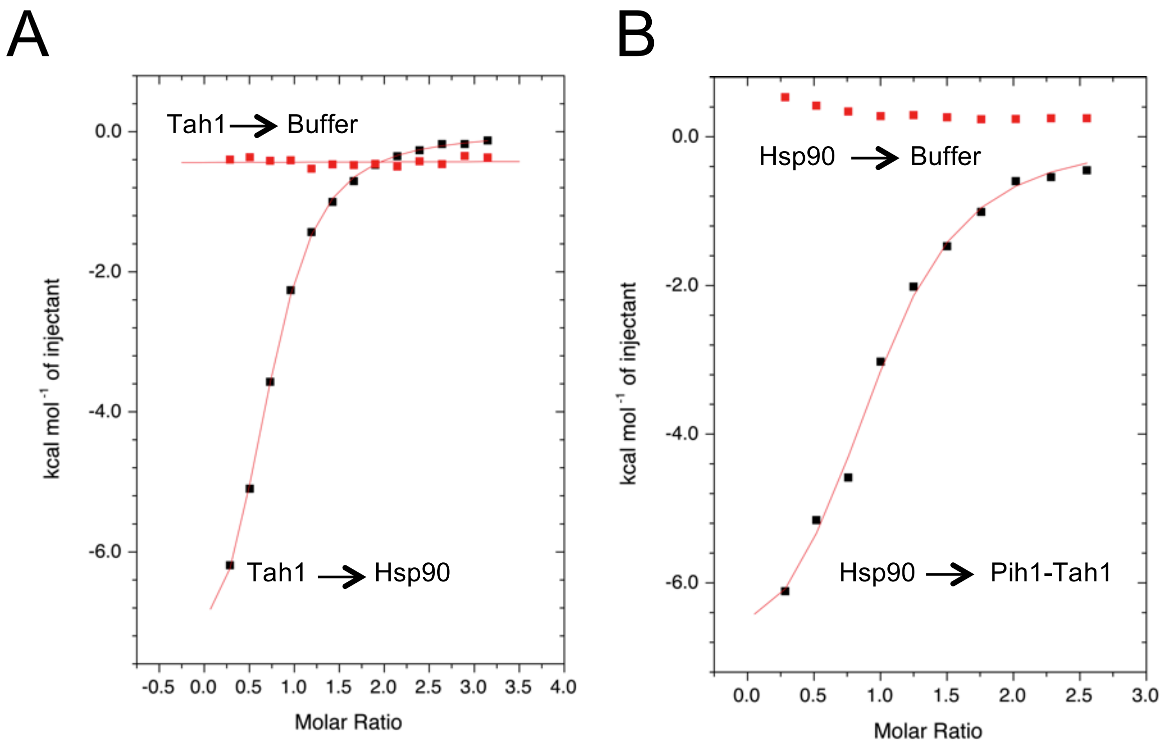

N = 0.68 ± 0.01 sites
K_D = 5.9 ± 0.30 μM
ΔH = -8445 ± 164.2 cal/mol
ΔS = -3.95 cal/mol/deg

N = 0.93 ± 0.04 sites
K_D = 4.0 ± 0.07 μM
ΔH = -7595 ± 404 cal/mol
ΔS = -0.367 cal/mol/deg

Pih1 interaction with Tah1 Dimer

A and B. Binding of Tah1 (**A**) or Tah1-Pih1 (**B**) to an Hsp90 dimer shows very similar affinity (K_D = 5.9 ± 0.3 μM, and K_D = 4 ± 0.07 μM, respectively) with a 1:1 stoichiometry (1 Hsp90 dimer: 2 Tah1) in both cases, confirming the possibility of each Tah1 domain bound to an Hsp90 dimer being able to recruit a Pih1 molecule.

Figure S2 – related to Figure 4

A

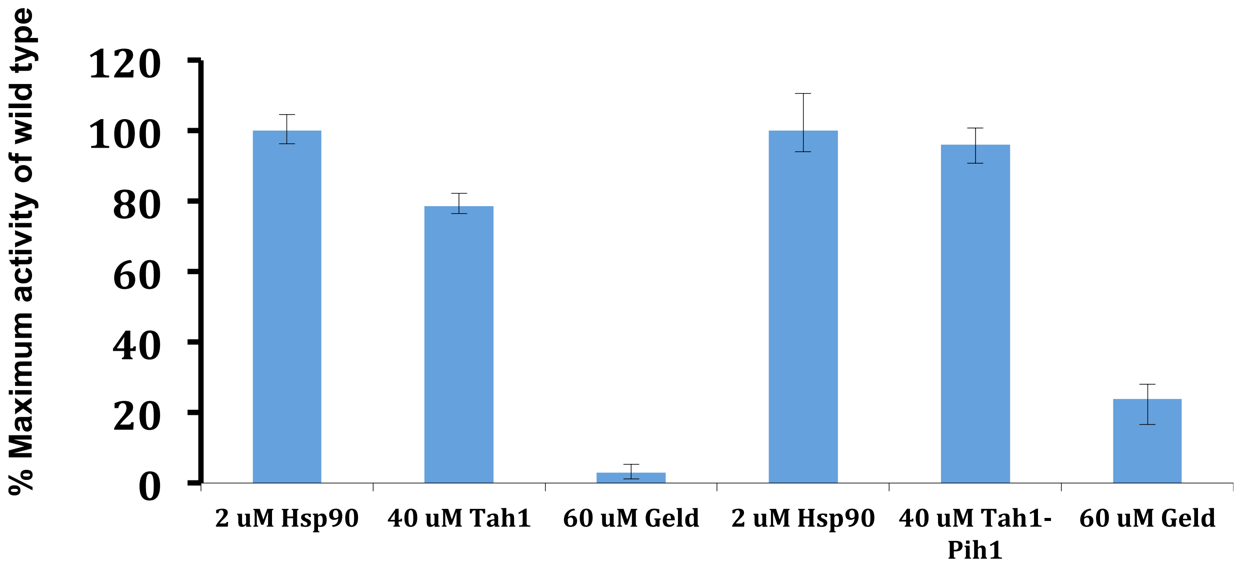

B

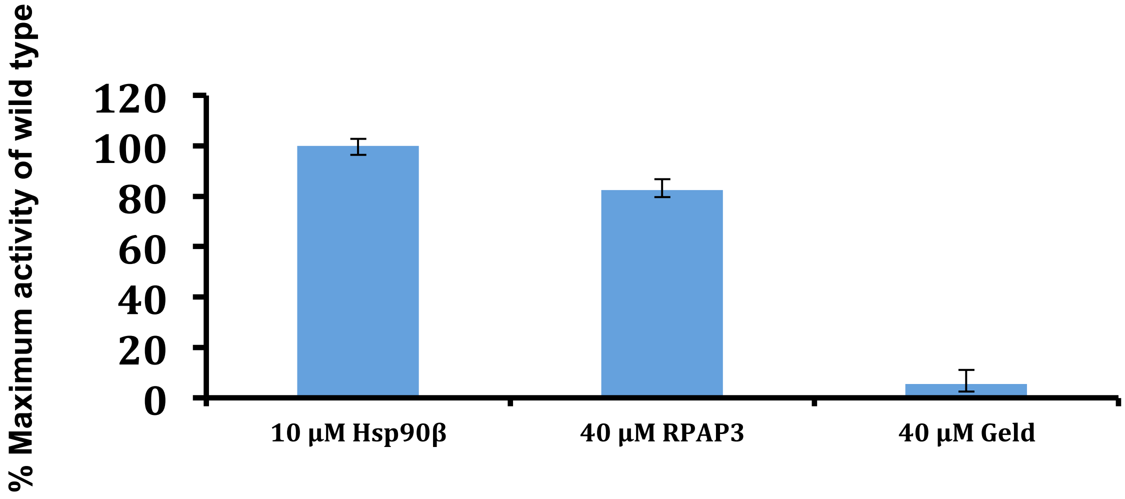

The effect of Tah1 and Spagh/RPAP3 binding on the ATPase activity of Hsp90

A. Binding of Tah1 and Tah1-Pih complex failed to show any strong regulatory effect on the ATPase activity of yeast Hsp90.

B. Binding of Spagh/RPAP3 failed to show any strong regulatory effect on the ATPase activity of human Hsp90β

Figure S3 – related to Figure 3

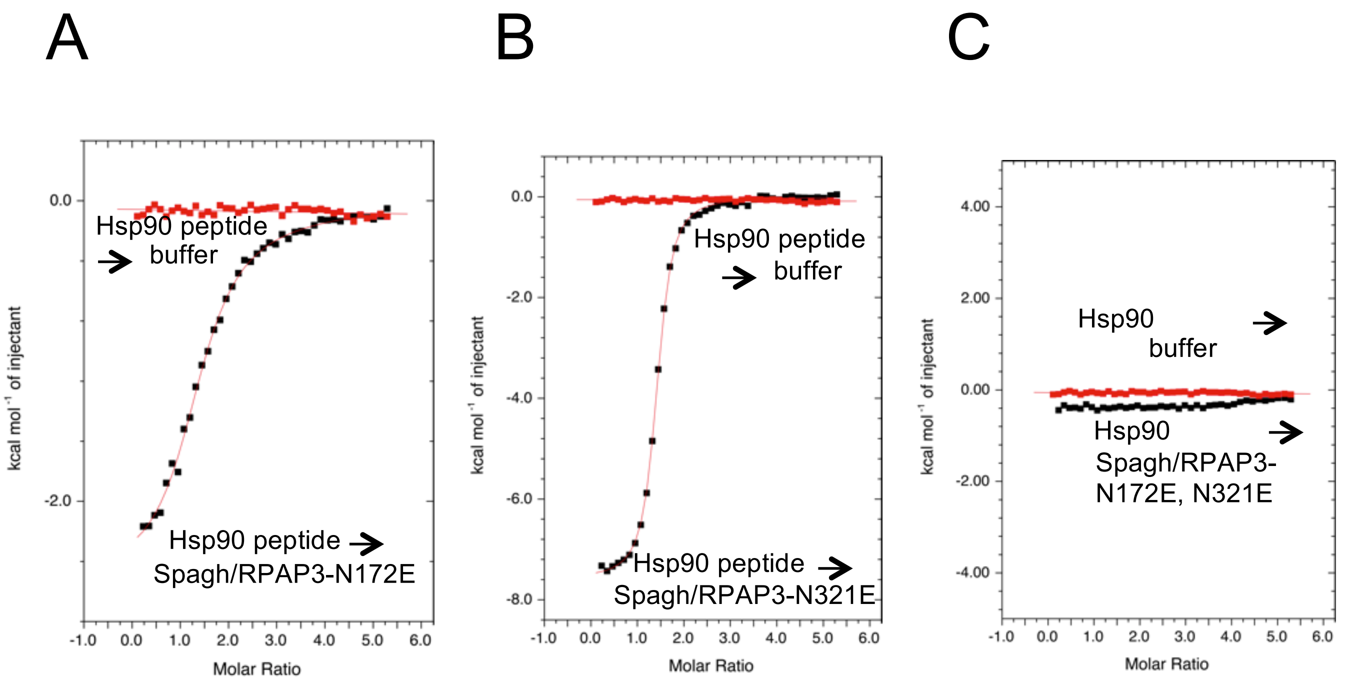

A	B
N1 = 1.36 ± 0.08 sites	N1 = 1.38 ± 0.02 sites
K_D1 = 9.7 ± 3.7 μM	K_D1 = 0.94 ± 0.94 μM
ΔH1 = -2500 ± 67.6 cal/mol	ΔH1 = -7584 ± 33.1 cal/mol
ΔS1 = 14.7 cal/mol/deg	ΔS1 = 2.55 cal/mol/deg
N2 = 1 site	N2 = 1 site
K_D2 = 231 ± 242 μM	K_D2 = 109.2 ± 270 μM
ΔH2 = -940.1 ± 172 cal/mol	ΔH2 = -100 ± 0.0 cal/mol
ΔS2 = 13.5 cal/mol/deg	ΔS2 = 17.8 cal/mol/deg

Binding of Hsp90 C-terminal peptide to Spagh/RPAP3 TPR domain mutants.

A. ITC of Hsp90 peptide binding to Spagh/RPAP3-N172E – TPR1 mutant. The single un-mutated site in TPR2 binds the Hsp90 peptide with K_D = 9.7 μM.

B. ITC of Hsp90 peptide binding to Spagh/RPAP3-N321E – TPR2 mutant. The single un-mutated site in TPR1 binds the Hsp90 peptide tightly with estimated K_D = 0.94 μM.

C. No Hsp90 peptide binding is observed with the Spagh/RPAP3-N172E, N321E double mutant.

Figure S4 – related to Figure 4

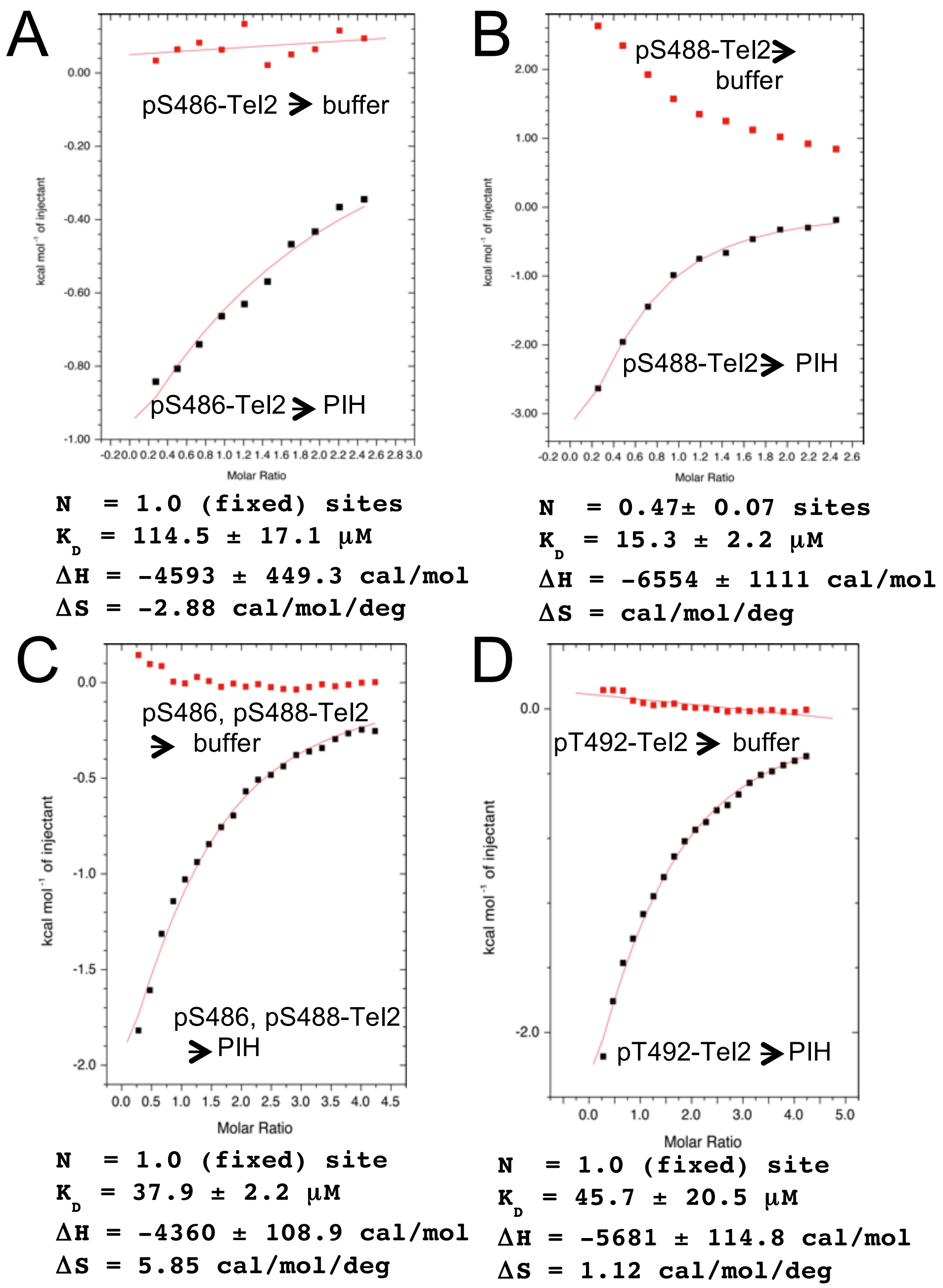

Binding of Tel2-peptides incorporating observed CK2 phosphorylation sites

A. ITC of binding of Tel2 peptide phosphorylated on Ser 486 to mouse Pih1-PIH domain. The peptide binds weakly with estimated $K_D \sim 114.5\ \mu M$.

B. ITC of binding of Tel2 peptide phosphorylated on Ser 488 to mouse Pih1-PIH domain. The peptide binds with $K_D = 15.3\ \mu M$.

C. ITC of binding of Tel2 peptide phosphorylated on Ser 486 and Ser 488 to mouse Pih1-PIH domain. The peptide binds with $K_D = 37.9\ \mu M$.

D. ITC of binding of Tel2 peptide, where phosphorylated Ser 492 is substituted for phosphorylated Thr 492, to mouse Pih1-PIH domain. The peptide binds with $K_D = 45.7\ \mu M$.

Figure S5 – related to Figure 5

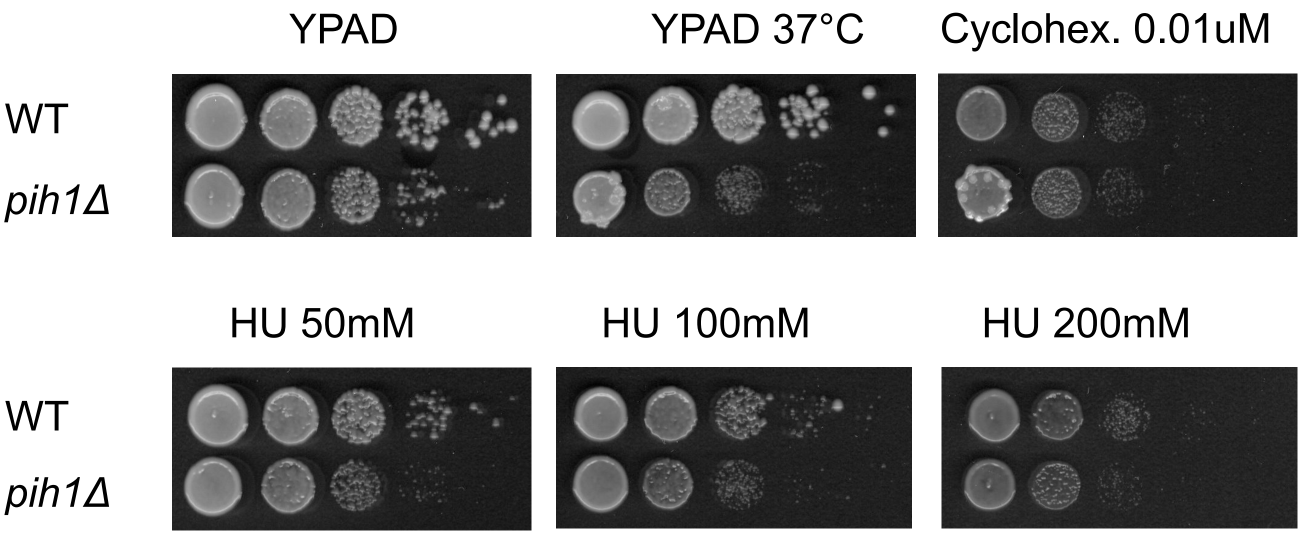

Pih1 and DNA damage sensitivity.

In contrast to the significant temperature sensitivity generated by the disruption of R2TP and TTT function, deletion of Pih1p does not confer any sensitivity to the DNA damaging agent hydroxyurea, compared to wild-type (WT).

Figure S6 – related to Figure 4

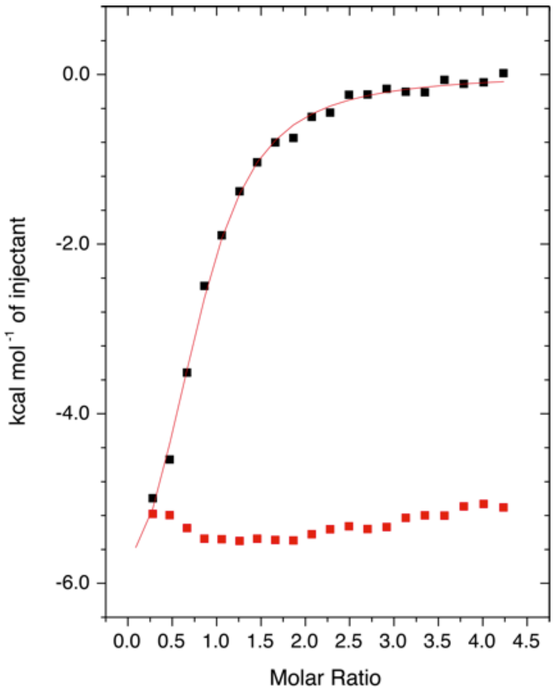

$N = 0.76 \pm 0.03$ sites
$K_D = 6.4 \pm 0.7\ \mu M$
$\Delta H = -7313 \pm 354.7$ cal/mol
$\Delta S = -0.36$ cal/mol/deg

The binding of the phosphoserine peptide of Mre11

A. Binding of the Mre11 phosphoserine peptide, MANDpSDDSI, to the PIH domain of Pih1. The peptide bound with a $K_D = 6.4\ \mu M$.
